# Optimized Magnesium Force Field Parameters for Biomolecular Simulations with Accurate Solvation, Ion-Binding, and Water-Exchange Properties

**DOI:** 10.1021/acs.jctc.0c01281

**Published:** 2021-03-15

**Authors:** Kara K. Grotz, Sergio Cruz-León, Nadine Schwierz

**Affiliations:** Department of Theoretical Biophysics, Max-Planck-Institute of Biophysics, Frankfurt am Main 60438, Germany

## Abstract

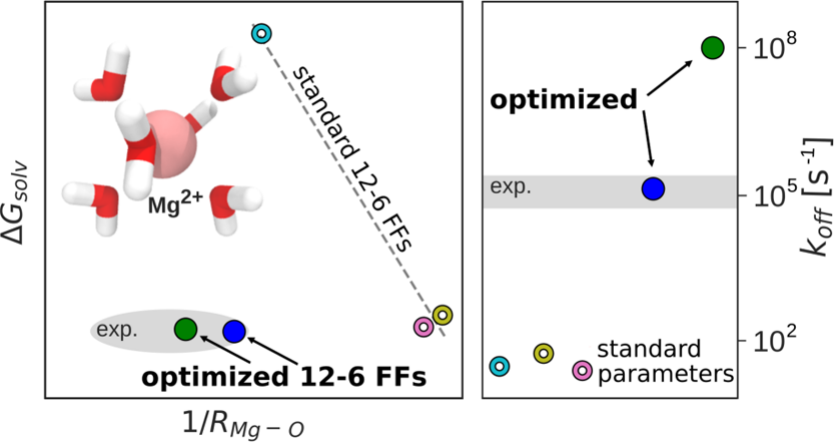

Magnesium ions play an essential role in many vital processes. To correctly describe their interactions in molecular dynamics simulations, an accurate parametrization is crucial. Despite the importance and considerable scientific effort, current force fields based on the commonly used 12–6 Lennard-Jones interaction potential fail to reproduce a variety of experimental solution properties. In particular, no parametrization exists so far that simultaneously reproduces the solvation free energy and the distance to the water oxygens in the first hydration shell. Moreover, current Mg^2+^ force fields significantly underestimate the rate of water exchange leading to unrealistically slow exchange kinetics. In order to make progress in the development of improved models, we systematically optimize the Mg^2+^ parameters in combination with the TIP3P water model in a much larger parameter space than previously done. The results show that a long-ranged interaction potential and modified Lorentz–Berthelot combination rules allow us to accurately reproduce multiple experimental properties including the solvation free energy, the distances to the oxygens of the first hydration shell, the hydration number, the activity coefficient derivative in MgCl_2_ solutions, the self-diffusion coefficient, and the binding affinity to the phosphate oxygen of RNA. Matching this broad range of thermodynamic properties, we present two sets of optimal parameters: *MicroMg* yields water exchange on the microsecond timescale in agreement with experiments. *NanoMg* yields water exchange on the nanosecond timescale facilitating the direct observation of ion-binding events. As shown for the example of the *add* A-riboswitch, the optimized parameters correctly reproduce the structure of specifically bound ions and permit the de novo prediction of Mg^2+^-binding sites in biomolecular simulations.

## 1 Introduction

Magnesium ions play a crucial role in a large variety of physiological processes such as ATP hydrolysis, cellular signaling, or the catalytic activity of enzymes and ribozymes.^1−4^ In particular, in nucleic acid systems, Mg^2+^ ions are essential to stabilize the tertiary structure, to drive folding or to enable catalytic reactions.^3,5−11^ Due to the biochemical importance of Mg^2+^, the modeling of these ions has received significant scientific attention.^12−21^ However, providing a quantitative description of their interactions and resolving their role in the folding and function of biomolecules is challenging. On the one hand, ab initio quantum mechanical approaches could provide unbiased insights but are limited to small system sizes. On the other hand, classical all-atom simulations allow us to treat much larger and biologically relevant systems but require accurate empirical force fields. Currently, in the most widely used force fields, Mg^2+^ ions are modeled as point charges and the electrostatic, dispersion, and excluded volume interactions are taken into account by a pairwise interaction potential. Hereby, the most common form of the nonelectrostatic interactions is the 12–6 Lennard-Jones (LJ) potential.^22^

In order to provide accurate Mg^2+^ models, the two parameters of the LJ potential are typically adjusted to reproduce experimental solution properties such as the solvation free energy Δ*G*_solv_,^17−20^ the distance to the water oxygens in the first hydration shell *R*_1_,^13,14,16,18,19^ and the coordination number *n*_1_.^13,16−20^ In addition to thermodynamic and structural data, including kinetic properties in the parametrization is crucial to capture dynamical processes such as water exchange, ion-binding, or ion-pairing.^16,20,23^ However, including kinetic data in the optimization is quite demanding since water exchange is on the microsecond timescale^24,25^ and involves the concerted motion of two exchanging water molecules.^26^ Therefore, simple and computationally efficient methods based on transition state theory are insufficient to provide an accurate rate estimate.^26^

Moreover, force fields optimized based on ion–water properties alone frequently fail to reproduce thermodynamic and structural properties of electrolytes at nonvanishing salt concentrations.^27,28^ It is therefore essential to balance ion–water and ion–ion interactions by including experimental data for activity coefficient derivatives or osmotic pressures in the optimization.^28−32^

Finally, in order to improve the applicability of the parameters for biomolecular simulations, it has proven useful to consider the interactions between Mg^2+^ and specific ion-binding sites on RNA and proteins.^33,34^

Reproducing this broad range of structural, thermodynamic, and kinetic properties by optimizing the force field parameters is tremendously challenging. Despite considerable scientific effort, none of the Mg^2+^ force fields from the literature based on the common 12–6 LJ interaction potentials is able to reproduce all properties with sufficient accuracy.^23^ For example, apparently, no parameter combination of the LJ parameters exists that simultaneously reproduces Δ*G*_solv_ and *R*_1_.^17,18,20,35^ In addition, current force fields underestimate the experimental rate of water exchange by several orders of magnitude leading to unrealistically slow exchange dynamics in biomolecular simulations.^20,23,26^ The too slow exchange kinetics has severe consequences since it governs any type of reaction involving the replacement of strongly bound hydration water. Therefore, important biochemical processes such as the transition from outer-to-inner sphere binding, chemical reactions in metalloenzymes or ribozymes, or the transport of ions across cell membranes^24,25,36−39^ become so rare that they cannot be simulated with sufficient statistics or, in many cases, not at all. In order to address this problem, Allnér et al. developed a set of Mg^2+^ parameters that reproduces the experimental exchange rate.^16^ However, similar to the Δ*G*_solv_–*R*_1_ parametrization problem, the authors failed to simultaneously reproduce the solvation free energy and the water exchange rate.

The aim of our current work is to provide optimized Mg^2+^ parameters in combination with the TIP3P water model that accurately reproduce all the abovementioned thermodynamic and kinetic properties. However, due to the complexity of the optimization problem, the question arises whether simple 12–6 potentials are sufficient for an accurate description or whether additional terms in the interaction potential are required. In particular, classical nonpolarizable force fields do not include many-body quantum effects explicitly. While Mg^2+^ ions themselves have a low polarizability, they polarize their environment strongly. Therefore, charge-induced dipole interactions and charge transfer can become significant, rendering the interaction between the cation and water more attractive and long-ranged compared to ions with low charge density. It therefore seems appealing to include additional parameters in the interaction potential to account for such polarization effects explicitly. For example, 12–6–4 potentials include an additional *r*^–4^ term that mimics the charge-induced dipole interactions.^19^ This parametrization strategy has proven successful in simultaneously reproducing Δ*G*_solv_, *R*_1_, and *n*_1_ for different metal ions.^19^ Another possibility is to modify the description of the electrostatic term.^40,41^ For example, Jungwirth and co-workers, introduced an additional charge scaling term to reproduce structural properties of aqueous MgCl_2_ from neutron-scattering experiments.^15^ Nevertheless, it is clear that any model with more parameters is expected to be in general more accurate if optimized properly with respect to all its parameters.

Here, we follow an alternative approach to improve the agreement with experimental properties without introducing more complex force fields. This approach is motivated by the fact that nonpolarizable force fields of the 12–6 type take polarizability into account implicitly. Therefore, instead of enlarging the parameter space of the interaction model, we explore the already existing force field parameter space in all depth. This includes an extensive optimization in an extended range of possible ion–water LJ parameters and a systematic optimization of the ion–ion and ion–RNA combination rules. Our optimization strategy is justified a posteriori by illustrating that the agreement with experimental results is similar or better compared to more complex force fields with additional terms in the interaction potential.

Our optimization procedure is done in three consecutive steps. In the first step, we optimize the ion–water interactions by selecting parameter combinations that reproduce Δ*G*_solv_, *R*_1_, and *n*_1_. In the second step, we optimize the water-exchange kinetics by choosing the combination that reproduces the experimental water-exchange rate (*microMg*). In addition, we choose a second parameter set that yields accelerated water-exchange dynamics (*nanoMg*). In the last step, we optimize the ion–ion and ion–biomolecule interactions by introducing scaling factors in the combination rules.^30^ This allows us to reproduce the activity derivative of MgCl_2_ solutions over a broad concentration range. Furthermore, we balance the Mg^2+^–RNA interactions by tuning the pairwise interaction to reproduce the binding affinity toward the nonbridging oxygen atoms of the phosphate group on RNA. Finally, we test the performance of our optimized parameters for the *add* A-riboswitch. *MicroMg* leads to stable RNA structures and correctly reproduces the structure of two specifically bound ions. This parameter set is particularly suited to simulate Mg^2+^ in aqueous solutions and its interactions with biomolecules such as nucleic acids, proteins, and lipids. On the other hand, *nanoMg* yields accelerated water-exchange dynamics and is therefore particularly suited to investigate specific ion-binding including the de novo prediction of inner-sphere ion-binding sites on RNA.

## 2 Methods

### 2.1 Molecular Dynamics Simulations

In the following, Mg^2+^ ions are modeled as point charges and the electrostatic, dispersion, and excluded volume interactions are taken into account by a pairwise interaction potential. Hereby, the most common form of the LJ potential is used with a repulsive *r*^–12^ and an attractive *r*^–6^ term. Overall, the interaction potential has the following form
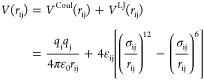
1where *q*_i_ and *q*_j_ are the charges of atoms i and j, respectively, *r*_ij_ is the distance between them, and ϵ_0_ is the dielectric constant of vacuum. The parameters σ_ij_ and ε_ij_ describe the LJ diameter and interaction strength, respectively. We refrain from adjusting parameters of the Coulomb term while the two parameters of the LJ potential are free to be optimized. The Lorentz–Berthelot combination rules are used to describe the interactions between atoms i and j
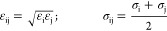
2

We used the TIP3P water model^42^ with LJ parameters of σ_o_ = 0.315061 nm and ε_o_ = 0.6364 kJ/mol. This choice was motivated by the fact that this water model is frequently used in biomolecular simulations since the AMBER force fields for nucleic acids and proteins have been optimized in combination with TIP3P water.

In order to compare our newly optimized parameters (Table 1) to force fields from the literature, we performed simulations using the 12–6-based parameters by Allnér–Villa,^16^ Mamatkulov–Schwierz,^20^ and Li–Merz^18^ (HFE set) and the 12–6–4-based parameters by Li–Merz.^19^ For our optimization procedure and for simulations with the Mg^2+^ parameters by Mamatkulov–Schwierz,^20^ Cl^–^ parameters were taken from Mamatkulov–Schwierz.^20^ In all other cases, the Cl^–^ parameters were taken from Joung–Cheatham.^43^ The parameters of all force fields used are listed in Table S6. Simulations with force fields of the 12–6 type were performed with GROMACS^44^ (versions 5.1.4, 2018, 2020). Simulations with force fields of the 12–6–4 type were performed with AMBER^45^ (version 2020) since GROMACS does not support 12–6–4 interaction potentials. An overview over the various simulation setups can be found in Section S1.1 (Table S1).

**Table 1:** Optimized Force Field Parameters for Mg^2+^ for Simulations with the TIP3P Water Model^a^

		*microMg*	*nanoMg*
σ_ii_	[nm]	0.1019	0.1025
ε_ii_	[kJ/mol]	235.80	389.80
σ_io_	[nm]	0.2085	0.2088
ε_io_	[kJ/mol]	12.250	15.750
λ_σ_^Cl^		1.8000	1.8000
λ_ε_^Cl^		0.1000	0.1000
σ_MgCl_	[nm]	0.4878	0.4884
ε_MgCl_	[kJ/mol]	0.8181	1.0518
λ_σ_^RNA^		1.1375	1.1435
λ_ε_^RNA^		0.3200	0.2500
σ_MgOP_	[nm]	0.2262	0.2277
ε_MgOP_	[kJ/mol]	4.6061	4.6266

a σ_ii_, ε_ii_, σ_io_, and ε_io_ are the ion–ion and ion–water LJ parameters. λ_σ_^X^ and λ_ε_^X^ are the scaling factors for the Lorentz–Berthelot combination rules (eq 3) for the interaction with Cl^–^ or the RNA atoms, shown exemplary for the interaction between Mg^2+^ and OP. Note that the scaling factors are only valid in combination with the Cl^–^ parameters from ref (20) and the parmBSC0χ_OL3_ RNA parameters.^50−52^

Following the work by Fyta and Netz,^30^ we introduce adjustable scaling parameters λ_σ_^X^ and λ_ε_^X^ in the Lorentz–Berthelot combination rules to describe the Mg^2+^–Cl^–^ and the Mg^2+^–RNA interactions. With this, the Lorentz–Berthelot combination rules have the following form
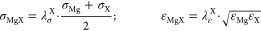
3where X denotes Cl^–^ or the atoms of the RNA. Note that these additional scaling factors leave the ion–water interaction parameters unchanged. Therefore, the solvation free energy, the structural properties of the first hydration shell, and the rate of water exchange remain unchanged.

To optimize the ion–RNA interactions, dimethylphosphate (DMP) was used similar to previous work.^16,33,46,47^ The DMP molecule contains two nonbridging phosphate oxygen atoms that are considered to be the most important inner-sphere Mg^2+^-binding sites. The force field parameters for the DMP molecule are based on a parametrization with GAFF^48^ (see Section S1.2).

For the *add* A-riboswitch (PDB ID: 1Y26(49)), the parmBSC0χ_OL3_ force field^50−52^ was used. Adenine was parameterized using GAFF (see Section S1.2).

The analysis was performed with the built-in GROMACS^44^ code and using the MDAnalysis package^53,54^ for python.

### 2.2 Optimization Procedure

In the first step, we optimize the ion–water interaction by performing a grid search in σ_io_–ε_io_ space. Initially, all σ_io_–ε_io_ parameter combinations are selected that match the experimental solvation free energy Δ*G*_solv_, the Mg^2+^–oxygen distance in the first hydrations shell *R*_1_, and the coordination number *n*_1_.

In the second step, we optimize the water-exchange dynamics by calculating the rate of water exchange for the abovementioned parameter combinations. Two parameter sets were chosen: the *microMg* parameter set yields water exchange on the microsecond timescale and reproduces the experimental rate exactly. The *nanoMg* parameter set yields water exchange on the nanosecond timescale, thereby providing exchange dynamics that are 2 orders of magnitude faster while still reproducing thermodynamic and structural properties.

In the last step, we optimize the ion–ion and ion–RNA interactions by calculating activity coefficient derivatives and ion-binding affinities by performing a grid search in λ_σ_^X^ and λ_ε_^X^ parameter space (eq 3). In particular, we used Kirkwood–Buff theory^55^ to calculate the activity coefficient derivatives *a*_cc_ and to select the scaling factors λ_σ_^Cl^ and λ_ε_^Cl^ that reproduce the experimental activity derivative over a broad range of MgCl_2_ concentrations. Subsequently, alchemical transformation calculations were used to calculate the binding affinity of Mg^2+^ toward one of the nonbridging phosphate oxygens of DMP. Finally, the scaling factors λ_σ,ε_^RNA^ that reproduce the experimental binding affinity Δ*G*_b_^0^ and binding distance *R*_b_ toward the phosphate oxygen were selected.

### 2.3 Free-Energy Perturbation and Single-Ion Properties

The solvation free energy of neutral MgCl_2_ ion pairs Δ*G*_solv_ was calculated following the same procedure described in our previous work.^20^ Since the proton solvation free energy used for absolute solvation free energies can vary according to different sources, we use the more robust solvation free energy of neutral MgCl_2_ ion pairs.^35^ The parameter range used in our current study is σ_io_ = 0.16–0.24 nm and ε_io_ = 1.8–28 kJ/mol. Finite size, pressure, and surface effect corrections were applied and simulations with three different box sizes yielded the same result in agreement with previous findings.^35,56^ Further details can be found in refs (20, 35) and Section S1.3.

In addition to Δ*G*_solv_, *R*_1_, and *n*_1_, the self-diffusion coefficient *D*_0_ was calculated (see Section S1.4).

### 2.4 Umbrella Sampling

One-dimensional free-energy profiles were calculated using umbrella sampling.^57,58^ The distance between Mg^2+^ and the leaving water molecule *r*_MgOw_1__ or between Mg^2+^ and one of the two nonbridging phosphate oxygens of DMP *r*_MgOP_ was used as the umbrella coordinate. Note that parameters defining partial charge, atom type, and angle potential of the two nonbridging phosphate oxygens of the DMP were adjusted to be identical to the AMBER RNA force field parameters (parmBSC0χ_OL3_,^50−52^ see Section S1.2). The Mg^2+^ parameters and scaling factors are therefore directly transferable.

The two-dimensional free-energy profile was calculated as a function of the distance between Mg^2+^ and the two exchanging water molecules, *r*_MgOw_1__ and *r*_MgOw_2__. During the sampling, additional restraints have been applied (see ref (26) and Section S1.5).

### 2.5 Rate Constant of Water Exchange

The rate constant *k* of water exchange in the first hydration shell of Mg^2+^ is defined by^24^
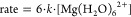
4where 6 is the coordination number of the first hydration shell and [Mg(H_2_O)_6_^2+^] is the concentration of hexa-coordinated Mg^2+^ ions.

The most popular theory to calculate reaction rates is transition state theory (TST).^59,60^ In simple systems for which the reaction coordinate is exactly known, TST gives an accurate estimate of the rate. However, in complex many-body systems as the ones presented here, TST can fail due to the violation of the non-recrossing hypothesis which forms the cornerstone of the theory. Therefore, in the following, we use TST only to compare to results from the literature or to provide an upper estimate for the rate constant (see Sections S1.6 and S2.3).

In order to provide an accurate rate estimate, we use 1 μs long trajectories of a 1 M MgCl_2_ solution and calculate the rate from the number of transitions over time. Hereby, we follow each water molecule individually through the trajectory. The rate constant *k* is then given by
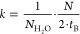
5where *N*_H_2_O_ is the number of water molecules in the simulation box and *N* is the total number of transitions for all water molecules (counting the exchange from first to second hydration shell and the reverse transition as individual events). *t*_B_ = *N*_Mg_ × *p*_B_ × *t*_sim_ is the cumulative time the water molecule spends in the first hydration shell of any Mg^2+^ ion. *N*_Mg_ is the number of Mg^2+^ ions in the simulation box, *p*_B_ = 6/(*N*_H_2_O_ – 6) is the probability of water to be in the first hydration shell, and *t*_sim_ is the total simulation time. The number of transitions is calculated from an indicator function which defines the bound and unbound state using two cutoff parameters. Different values for the cutoff parameters were tested and the calculated rates were found to be insensitive to the exact definition (for further details see Section S1.7). Errors are calculated from block averaging^61^ by dividing the trajectory into two blocks.

### 2.6 Kirkwood–Buff Theory

To optimize the scaling factors for ion–ion interactions, 150 ns long simulations were performed at finite salt concentration. The parameter range investigated was λ_σ_^Cl^ = 1–2.6 and λ_ε_^Cl^ = 0.01–1. The activity coefficient derivatives *a*_cc_ were calculated using Kirkwood–Buff theory.^55^ The optimization was done for a concentration of 0.25 M MgCl_2_. Additional simulations at MgCl_2_ concentrations of 0.25, 0.5, 1, and 2 M were performed for the final parameter sets. Errors were calculated from dividing the trajectories into three blocks and block averaging. Further details on the calculation of *a*_cc_ can be found in Section S1.8.

### 2.7 Alchemical Transformation

To optimize the scaling factors for ion–RNA interactions, the binding affinity was calculated from alchemical transformations (see Section S1.9 for simulation details). In particular, the binding affinity Δ*G*_b_^0^ and the binding distance *R*_b_ toward one of the nonbridging phosphate oxygens of DMP were calculated for the parameter range λ_σ_^RNA^ = 0.97–1.23 and λ_ε_^RNA^ = 0.08–1.04. Subsequently, the scaling factors that reproduced the experimental value for Δ*G*_b_^0^^62^ and *R*_b_(63) were selected. Note that in the experimental work by Sigel and Sigel,^62^ two sets of values are given for the binding affinity. The first value is the stability constant of the DMP (log *K* = 0.45 or equivalently Δ*G*_b_^0^ = −1.036 *k*_B_*T*). The second value (log *K* = 1.05 or Δ*G*_b_^0^ = −2.418 *k*_B_*T*) is the stability constant of a modular RNA model. The value takes into account the fourfold access of the phosphate oxygen-binding site on the backbone compared to the nucleobase-binding sites and is appropriate only within the context of the modular RNA model. For our simulations, hence, the first value (log *K* = 0.45) is appropriate and was used in the optimization.

To further validate the results from alchemical transformations, Δ*G*_b_^0^ and *R*_b_ were calculated independently from free-energy profiles obtained for the final parameter sets (Section S1.9). Both methods yielded identical results within error (Section S2.4). Similarly, Δ*G*_b_^0^ and *R*_b_ for the Allnér–Villa and Panteva–York m12–6–4 parameters^33^ were calculated from free-energy profiles. The free-energy profiles were obtained from refs (16, 33) with permission.

Errors were calculated from block averaging by dividing the trajectory of the alchemical transformation into three blocks.

### 2.8 Performance of *MicroMg* and *NanoMg* for the *add* A-Riboswitch

To test the performance of our optimized parameter sets in a biologically relevant RNA system, the *add* A-riboswitch was simulated for 100 ns. The simulations included five Mg^2+^ ions positioned as observed in the X-ray structure (PDB ID: 1Y26,^49^ resolution: 2.10 Å). A total of 30 additional Mg^2+^ ions were placed randomly into the simulation box to neutralize the charge of the riboswitch. The rmsd was calculated discarding the first 2 ns for equilibration.

In a second setup, used to predict inner-sphere binding sites with *nanoMg*, 10 replicas of 200 ns were simulated. Here, all Mg^2+^ ions were placed randomly in the simulation box.

Three-dimensional Mg^2+^ densities were obtained with GROmaρs.^64^ The density was visualized with PyMOL.^65^

## 3 Results and Discussion

In the following, we present the results from our optimization procedure. The optimization is performed in three sequential steps and is aimed to capture ion–water, ion–ion, and ion–RNA interactions. In particular, the optimization is designed to simultaneously reproduce the solvation free energy, the distance to oxygens in the first hydration shell, the hydration number, the activity coefficient derivative in MgCl_2_ solutions, the self-diffusion coefficient, and the binding affinity and distance to the phosphate oxygens of RNA (Tables 2 and 3).

**Table 2:** Results for Single-Ion, Ion–Ion, and Ion–RNA Properties for the Optimized Parameters in Direct Comparison with Experimental Results^a^

	Δ*G*_solv_ [kJ/mol]	*R*_1_ [nm]	*n*_1_	*D*_0_ [10^–5^ cm^2^/s]	Δ*G*_b_^0^ [*k*_B_*T*]	*R*_b_ [nm]	*a*_cc_
*microMg*	–2532.9 ± 1	0.207 ± 0.004	6	0.754 ± 0.006	–0.633 ± 0.6	0.207 ± 0.004	0.93 ± 0.01
*nanoMg*	–2532.0 ± 1	0.209 ± 0.004	6	0.750 ± 0.004	–0.375 ± 0.1	0.207 ± 0.004	0.97 ± 0.01
exp.	–2532^79^	0.209 ± 0.004^66^	6^66^	0.706^79^	–1.036^62^	0.206–0.208^63^	0.93^80^

a Solvation free energy of neutral MgCl_2_ ion pairs Δ*G*_solv_, Mg^2+^–oxygen distance in the first hydration shell *R*_1_, coordination number of the first hydration shell *n*_1_, self-diffusion coefficient *D*_0_, binding affinity toward the phosphate oxygen of DMP ΔG_b_^0^, Mg^2+^–phosphate oxygen distance in the inner-sphere conformation *R*_b_, and *a*_cc_ is the activity derivative of a MgCl_2_ solution at 0.25 M concentration. Δ*G*_b_^0^ is derived from the log stability constant of the DMP (log *K* = 0.45) given in ref (62).

**Table 3:** Properties of Water Exchange from Simulations and Experiments^a^

	*N*	*k* [s^–1^]
*microMg*	376 ± 56	(8.04 ± 1.20) × 10^5^
*nanoMg*	52,086 ± 120	(1.11 ± 0.003) × 10^8^
Mamatkulov–Schwierz	2 ± 2	24.0 ± 8.8 from ref (26)
Allnér–Villa	2 ± 2	<2.4 × 10^5^
Li–Merz (12–6)	2 ± 2	<3.5 × 10^4^
Li–Merz (12–6–4)	6720 ± 160	(1.44 ± 0.03) × 10^7^
exp.	248,^25^ 314^24^	5.3 × 10^5^ from ref (24), 6.7 × 10^5^ from ref (25)

a Number of transitions *N* in 1 μs for different force fields in 1 M MgCl_2_ solutions. The experimental value^24,25^ is obtained from eq 5. The rate constant *k* is calculated from the number of transitions for *microMg*, *nanoMg*, and Li–Merz (12–6–4). The value for Mamatkulov–Schwierz is taken from ref (26). For Allnér–Villa and Li–Merz (12–6), an upper estimate is given based on TST since the number of transitions is insufficient to calculate the rate from eq 5. The errors for *N* and *k* are obtained from block averaging.

Matching this broad range of structural and thermodynamic properties, we present two sets of optimal parameters: *MicroMg* yields water exchange on the microsecond timescale and matches the experimental exchange rate. *NanoMg* yields water exchange on the nanosecond timescale. Subsequently, we validate the performance of our optimized parameter sets for the example of the *add* A-riboswitch.

### 3.1 Optimization of Solvation Free Energy, Mg^2+^–Water Distance, and Coordination Number

In the first step, we optimize the ion–water interactions by adjusting the LJ parameters σ_io_ and ε_io_ to reproduce the experimental solvation free energy Δ*G*_solv_, the distance to the oxygens in the first hydration shell *R*_1_, and the coordination number *n*_1_ (Figure S1). Since Δ*G*_solv_ includes the energy and entropy of ion hydration, it is considered the most important thermodynamic property in the development of accurate force field parameters.^35^ Moreover, Mg^2+^ is coordinated by six water molecules arranged in octahedral symmetry.^66^ In order to correctly capture the structure of the first hydration shell, we include *R*_1_ and *n*_1_ in our optimization.

**Figure 1:**
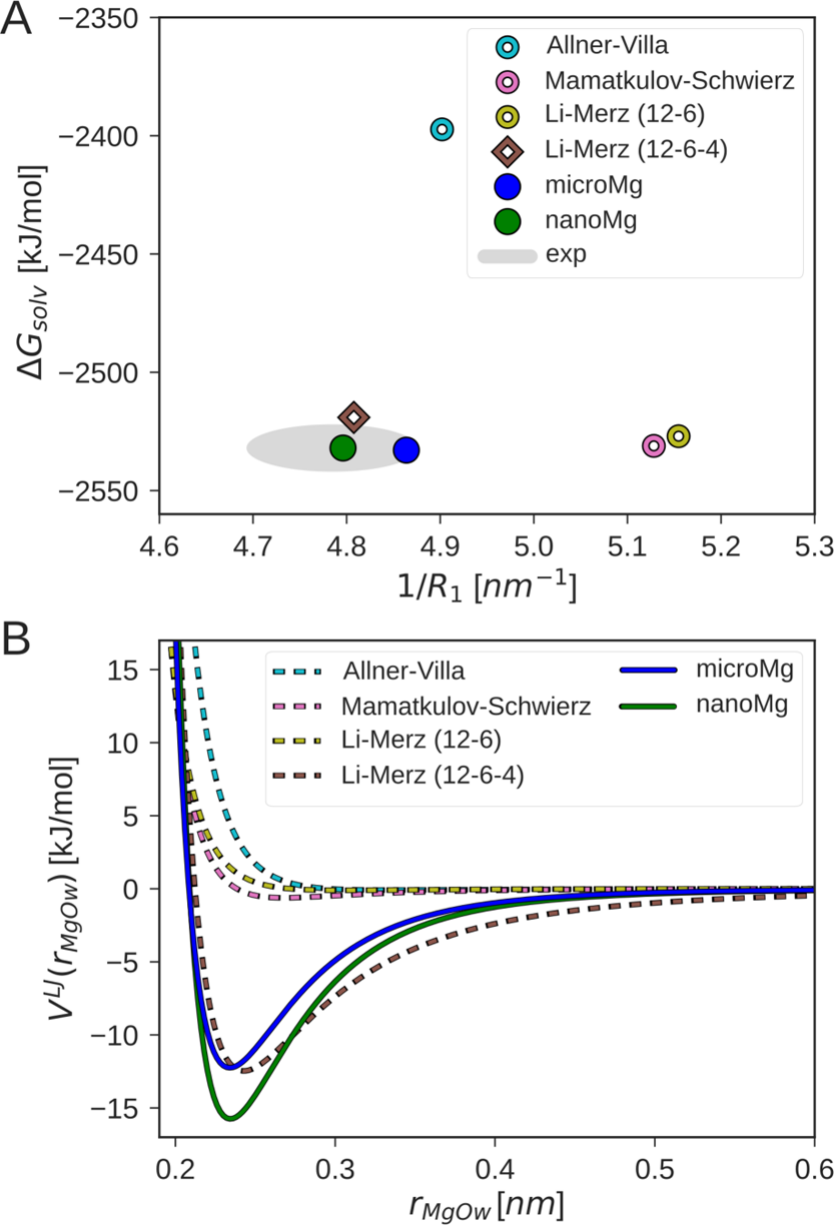
Comparison of the optimized parameter sets *microMg* and *nanoMg* with force fields from the literature^16,18−20^ and experimental data. (A) Solvation free energy Δ*G*_solv_ for neutral MgCl_2_ pairs in correlation with the inverse of the Mg^2+^–oxygen distance of the first hydration shell 1/*R*_1_. The gray area indicates the experimental results from refs (66, 70). (B) Lennard-Jones interaction potential *V*^LJ^ as a function of the Mg^2+^–oxygen distance *r*_MgOw_ for different Mg^2+^ force fields and TIP3P water.

Reproducing Δ*G*_solv_ and *R*_1_ with force fields of the 12–6 type is challenging. As illustrated in Figure 1A, in previous work, no parameter combination could be found that matches both properties simultaneously.^16−18,20,35^ In order to improve the agreement, we have considered a much larger σ_io_ and ε_io_ range in the optimization (Figure S1). As shown in Figures 1A and S1 and Table 2, this allows us to accurately reproduce Δ*G*_solv_, *R*_1_, and *n*_1_. Hereby, the agreement with experimental results is comparable to 12–6–4 interaction potentials which have one additional adjustable parameter. Based on the results for Δ*G*_solv_ and *R*_1_, we conclude that additional terms in the interaction potential that mimic polarization effects are not strictly necessary. Nevertheless, charge-induced dipole interactions and charge transfer are particularly important for Mg^2+^ ions in aqueous solutions. Both effects render the interaction potential more attractive and long-ranged compared to metal cations with lower charge density. This becomes evident from Figure 1B: the interaction potential of our optimized parameter sets *microMg* and *nanoMg* and the 12–6–4 potential by Li–Merz are similar in shape and more attractive and long-ranged compared to previous 12–6-based force fields. Capturing the long-ranged interactions is therefore crucial to correctly describe ion–water interactions in general and Δ*G*_solv_ and *R*_1_ in particular.

### 3.2 Optimization of the Water-Exchange Rate

In aqueous solutions, water molecules from the first tightly bound hydration shell around Mg^2+^ exchange with the second hydration shell on the microsecond timescale.^24,25^ In the second step of our parametrization, we optimize the water-exchange dynamics by calculating the rate constant *k* of water exchange in the range of parameter combinations that reproduce Δ*G*_solv_, *R*_1_, and *n*_1_ obtained in the previous optimization step. Hereby, special emphasis is placed on an accurate calculation of the rate constant. The reason is that the popular and frequently used transition state theory (TST) can fail due to the violation of the noncrossing hypothesis. In fact, recent work shows that for water exchange, TST based on the Mg^2+^–water distance overestimates the true rate by more than 2 orders of magnitude.^26^ In order to provide a more accurate rate estimate, we use 1 μs long simulations of 1 M MgCl_2_ solutions to calculate the rate from the number of transitions (eq 5). Based on this, two parameter combinations were selected (Table 3): the parameter set *microMg* yields water exchange on the microsecond timescale in agreement with experimental results.^24,25^ The parameter set *nanoMg* yields water exchange on the nanosecond timescale. Hereby, we chose the parameter combination that leads to the fastest exchange while still matching all other experimental properties.

**Figure 2:**
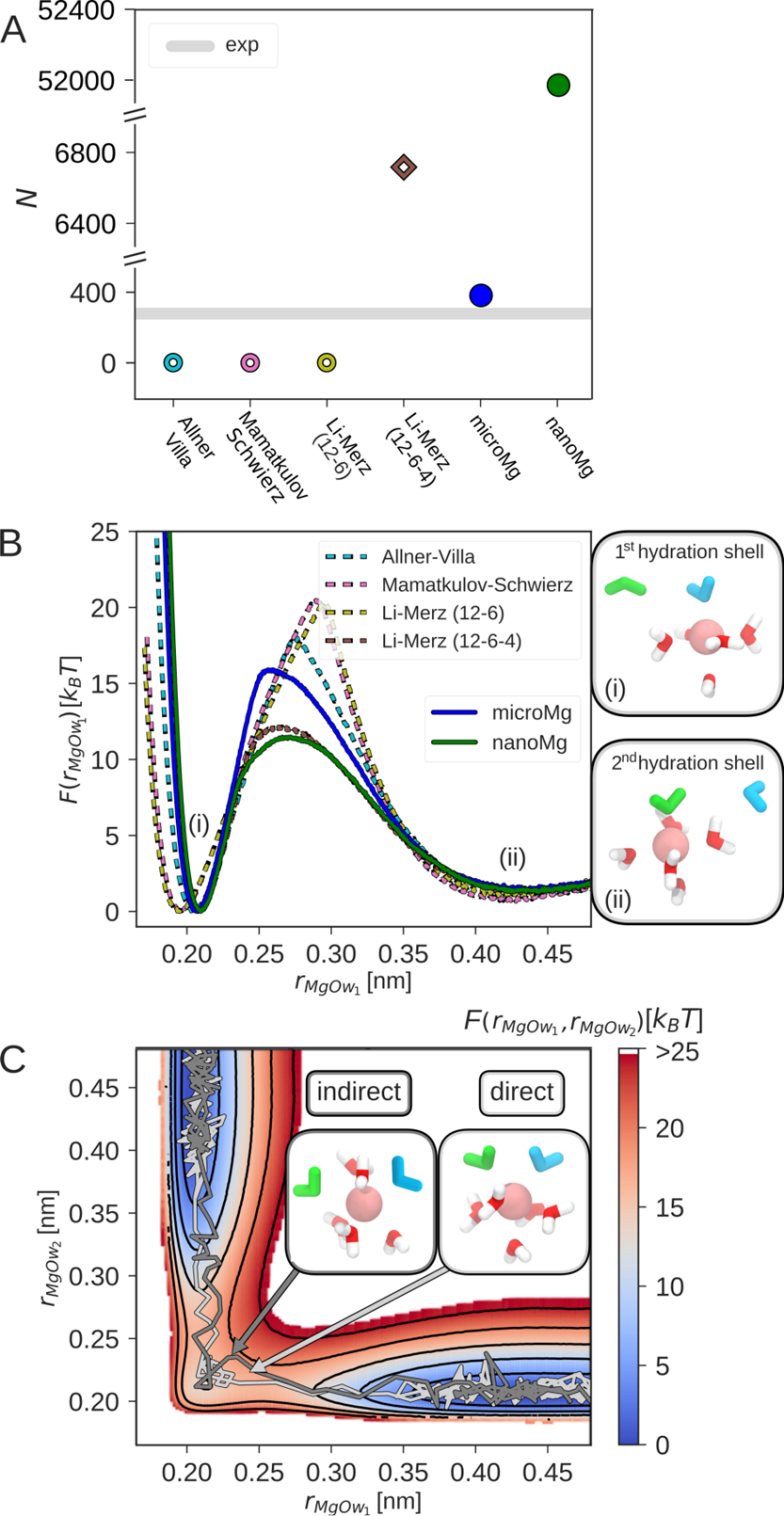
Water exchange in the first hydration shell of Mg^2+^. (A) Number of exchange events in 1 μs for different force fields in 1 M MgCl_2_ solutions. The gray horizontal line indicates the experimental values^24,25^ (Table 3). (B) One-dimensional free-energy profiles as a function of the distance between Mg^2+^ and the leaving water molecule *r*_MgOw_1__ for different force fields. The snapshots show representative conformations in the two stable states: (i) before exchange: leaving water (Ow_1_ shown in blue) is in the first hydration shell and incoming water (Ow_2_ shown in green) is in the second hydration shell. (ii) After exchange: Ow_1_ is in the second and Ow_2_ is in the first hydration shell. (C) Two-dimensional free-energy profile as a function of the distances between Mg^2+^ and the two exchanging waters, *r*_MgOw_2__ and *r*_MgOw_1__, obtained with the *microMg* parameters. The insets show representative snapshots of the transition state along the direct (dark gray) and the indirect (light gray) exchange pathway. The energy contour spacing corresponds to 5 *k*_B_*T*.

Figure 2A compares the number of exchanges for different force fields with the 248–314 transitions expected from experiments.^24,25^ The results reveal that water exchange is several orders of magnitude too slow for the force fields by Allnér–Villa,^16^ Mamatkulov–Schwierz,^20^ and Li–Merz (12–6).^18^ Surprisingly, the rate calculated from the Allnér–Villa parameters deviates from the experimental results despite being optimized on the exchange kinetics. The reasons are twofold: the Allnér–Villa parameters were optimized with the mTIP water model,^67^ and transferring the parameters to TIP3P leads to deviations (Section S2.2). In addition, the rate used in the optimization was calculated from TST and might therefore deviate notably from the true rate as discussed above. The number of exchanges for the Li–Merz 12–6–4 parameters is significantly higher and thus the rate overestimates experiments by 1 order of magnitude (Table 3). As expected from the current optimization procedure, the number of transitions for *microMg* closely matches the experimental value while *nanoMg* yields the highest number of exchanges of all force fields considered here.

Qualitative insights into the cause of the vastly different timescales of water exchange for the different force fields can be gained from the free-energy profiles along the Mg^2+^–water distance *r*_MgOw_1__ (Figure 2B). The free-energy profiles have two minima corresponding to the first and second hydration shell [shown in snapshots (i,ii) of Figure 2B]. The two minima are separated by a high free-energy barrier which corresponds to the cost of removing one water from the tightly bound first hydration shell. The height of the barriers differs by more than 10 *k*_B_*T* for the different force fields. Force fields with slow exchange kinetics [Li–Merz (12–6), Mamatkulov–Schwierz, and Allnér–Villa] have high energetic barriers of 20.4, 20.7, and 18.0 *k*_B_*T*. Force fields with comparatively faster exchange kinetics [*microMg*, Li–Merz (12–6–4), and *nanoMg*] have lower energetic barriers of 15.9, 12.2, and 11.5 *k*_B_*T*, respectively.

Water-exchange dynamics is, however, more complex than the one-dimensional free-energy profiles might suggest. It involves the concerted motion of two exchanging water molecules in which the molecular void provoked by the leaving water is immediately filled by an entering water molecule.^26^ In order to provide a more realistic picture, Figure 2C shows the two-dimensional free-energy profile as a function of the distance of the two exchanging waters *r*_MgOw_1__ and *r*_MgOw_2__ obtained with the *microMg* parameters (for *nanoMg* see Figure S3). Two exchange pathways are shown corresponding to a direct and indirect exchange mechanism in agreement with our previous results.^26^

In summary, the optimized *microMg* parameters yield much closer agreement with experimental water exchange rates compared to force fields from the literature (Table 3). In addition, the self-diffusion coefficient *D*_0_ matches the experimental value without further optimization (Table 2).

### 3.3 Optimization of Activity Derivative and Binding Affinity

**Figure 3:**
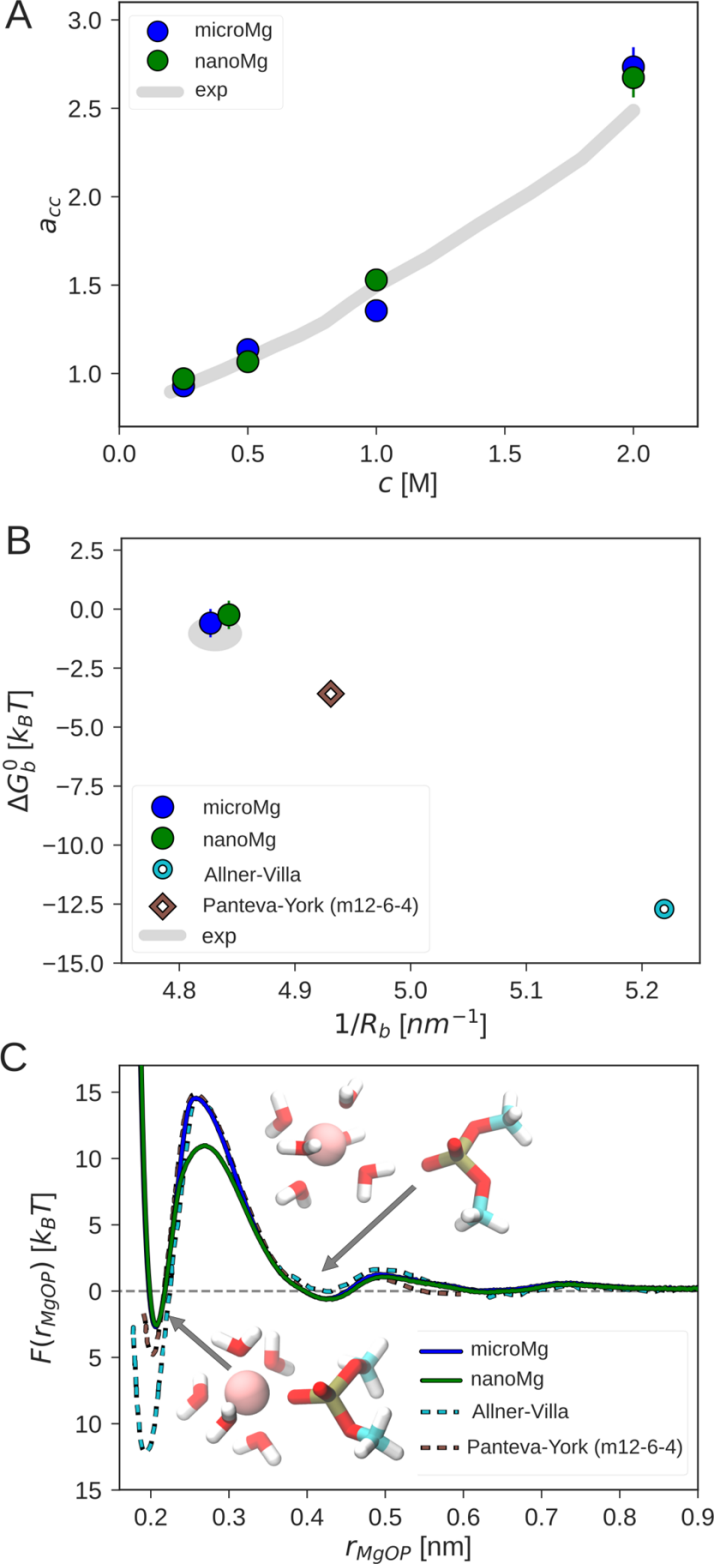
Optimization of ion–ion and the ion–RNA interactions. (A) Activity derivative *a*_cc_ as a function of the MgCl_2_ salt concentration with the optimized scaling factors for *microMg* and *nanoMg* (Table 1) and from experiments.^80^ (B) Binding affinity Δ*G*_b_^0^ in correlation with inverse of the Mg^2+^–phosphate oxygen distance 1/*R*_b_. The experimental values (gray) are taken from refs (62, 63). (C) Free-energy profiles as a function of the distance between Mg^2+^ and the phosphate oxygen of DMP, *r*_MgOP_, for different force fields. The Allnér–Villa and Panteva–York free-energy profiles were obtained from refs (16, 33) with permission. The insets show Mg^2+^ in the inner- and outer-sphere conformation.

In the last step of our parametrization, we sequentially optimize the ion–ion and the ion–RNA interactions. In order to correctly capture ion-pairing, a proper balance between ion–water and ion–ion interactions is required. This balance is achieved by optimizing the parameters based on experimental activity coefficients.^28^ Following the seminal work by Fyta and Netz,^30^ we introduce scaling parameters in the Lorentz–Berthelot combination rules (eq 3). The advantage of this approach is that it allows us to reproduce the experimental activity coefficient derivative *a*_cc_ over a broad concentration range for MgCl_2_ solutions (Figure 3A) without changing Δ*G*_solv_, *R*_1_, *n*_1_, *D*_0_, or *k*. In fact, with the standard combination rules (eq 2), *a*_cc_ was found to be too small for all parameter combinations investigated, in agreement with previous findings.^17,20,30^ Since no solid foundation for the standard combination rules exists, it is not surprising that they fail to capture cation–anion interactions. Targeting this interaction in the optimization therefore appears to be the natural choice. The resulting scaling factors (Table 1) reflect that without modifications, the Mg^2+^–Cl^–^ interactions are too attractive. This problem can easily be corrected by increasing the effective diameter of the outer-sphere ion pair via λ_σ_^Cl^ and by reducing the cation–anion LJ energy via λ_ε_^Cl^.

So far, the optimization was performed based on bulk ion properties. However, this does not guarantee that the interactions of Mg^2+^ and specific ion-binding sites on biomolecules are described correctly. For example, the 12–6–4 Li–Merz parameters significantly overestimate the Mg^2+^–RNA interactions.^33^ In order to solve this problem, modified m12–6–4 parameters were developed by Panteva and co-workers to reproduce experimental site-specific binding affinities.^33^

Here, we introduce ion–RNA scaling parameters in the Lorentz–Berthelot combination rules (eq 3) that are optimized based on experimental binding affinities^62^ and structural properties of the inner-sphere conformation.^63^ Similar to previous work,^16,33,46,47^ DMP was used. The DMP molecule contains two nonbridging phosphate oxygen atoms that are considered to be the most important inner-sphere Mg^2+^-binding sites on larger RNA molecules.^3,62,68,69^ Similar to *a*_cc_, the unmodified combination rules result in Mg^2+^–RNA interactions that are too attractive, possibly reflecting the small excess polarizability of Mg^2+^. Again, this can be corrected by increasing the effective diameter via λ_σ_^RNA^ and by reducing the cation–RNA LJ energy via λ_ε_^RNA^ (Table 1, Section S2.4).

Figure 3B summarizes the binding affinity Δ*G*_b_^0^ and binding distance *R*_b_ for different force fields from the literature. As expected, the Allnér–Villa parameters show the largest deviations from the experimental results. The Panteva–York m12–6–4 parameters provide significant improvement. Finally, the optimized ion–RNA scaling factors for *microMg* and *nanoMg* provide the closest agreement with experimental results.

Initial insights into the process of ion-binding to RNA is obtained from the free-energy profiles as a function of the distance between Mg^2+^ and the phosphate oxygen of DMP for different force fields (Figure 3C). The free-energy profiles have two stable states corresponding to the inner-sphere and the outer-sphere conformation (inset of Figure 3C). In the inner-sphere conformation, Mg^2+^ forms a direct contact with the phosphate oxygen of the DMP. In the outer-sphere conformation, the contact is mediated by a water molecule from the first hydration shell. There are clear deviations between the free-energy profiles from different force fields. The different depths of the first minimum reflect the different binding affinities as discussed above. Consequently, force fields that overestimate the binding affinity have a higher free-energy barrier and therefore slower dissociation kinetics. Similarly, the minimum in the free-energy profile is shifted to the left for force fields that underestimate *R*_b_.

The free-energy barrier for *nanoMg* is 3.5 k_B_T lower compared to *microMg* while Δ*G*_b_^0^ and *R*_b_ are identical. Removing a water molecule from the first hydration shell for *nanoMg* requires less work as reflected in the higher water-exchange rate (Table 3). Mg^2+^ association and dissociation is therefore 2–4 orders of magnitude faster compared to other force fields.

In summary, matching Δ*G*_b_^0^, *R*_b_, and the rate of water exchange is crucial to describe the kinetics of ion binding and the structure of specifically bound Mg^2+^ ions. In this respect, *microMg* is particularly suited to reproduce the distribution and exchange of Mg^2+^ as closely as possible. On the other hand, the ion-binding kinetics for *nanoMg* is significantly enhanced. This opens up the possibility to use *nanoMg* to predict inner-sphere ion-binding sites from straightforward simulations without the necessity to use enhanced sampling techniques.^70^

### 3.4 Performance of *MicroMg* and *NanoMg* for the *add* A-Riboswitch

**Figure 4:**
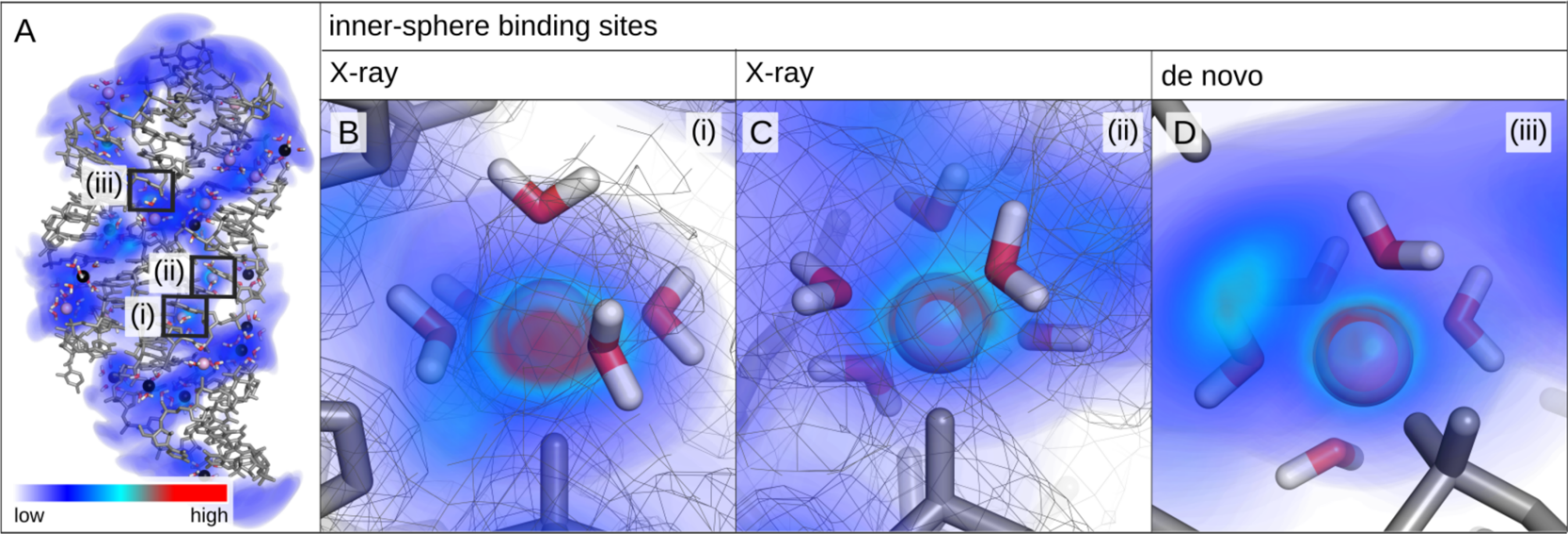
Representative snapshot of the *add* A-riboswitch from simulations with the *nanoMg* parameter set and three-dimensional Mg^2+^ probability density. The probability density is low in the blue regions (diffusive ions) and high in the red regions (specifically bound ions). Selected inner-sphere (pink) and outer-sphere (black) Mg^2+^ ions are shown including the water molecules in their first hydration shell. Snapshots (i–iii) show the most probable ion-binding sites predicted from simulations with *nanoMg*. Snapshots (i,ii) coincide with the two inner-sphere binding sites reported in the X-ray structure.^49^ The experimental electron density is shown as gray mesh.

In order to evaluate the performance of the optimized parameter sets, the *add* A-riboswitch was simulated (Figure 4A). This riboswitch is particularly suited to validate the parameters since experimental^49,71−73^ and simulation results^16,74^ exist.

The experimental X-ray structure^49^ includes five Mg^2+^ ions (PDB ID: 1Y26,^49^ resolution: 2.10 Å). Two ions are bound in inner-sphere conformation to phosphate oxygens, two ions are involved in outer-sphere interactions, while the remaining ion is a crystallization artifact due to crystal packing.

#### 3.4.1 Stability and Specific Ion-Binding Sites with *MicroMg* and *NanoMg*

Initially, we investigate that *microMg* and *nanoMg* do not affect the stability of the tertiary structure. In 100 ns simulations, both parameter sets yield stable structures that are close to the experimental structure with an rmsd of 0.28 (*microMg*) and 0.24 nm (*nanoMg*). Both values are slightly smaller compared to the rmsd of 0.30 nm obtained with the Allnér–Villa parameters.^16^

With *microMg*, both inner-shell ions of the X-ray structure remain bound over the duration of the simulation. The distance of Mg^2+^ to the phosphate oxygen of the first binding site (Figure 4B) is 0.208 ± 0.005 nm in close agreement with the 0.210 nm in the X-ray structure. The average distance of Mg^2+^ to the oxygen phosphate of the second binding site (Figure 4C) is 0.209 ± 0.005 nm. This value is smaller compared to the 0.244 nm in the X-ray structure. Note, however, that the experimental value is located in the exclusion range and might be over-rated as judged by the assignment criterion of 0.206–0.208 nm.^63^ With *nanoMg*, the distances are similar with 0.209 ± 0.012 nm for both binding sites. Here, however, the ions exchange with bulk due to the faster exchange kinetics discussed above. After 100 ns, 9 Mg^2+^ ions are observed in inner-sphere conformation with nonbridging phosphate oxygens. No inner-sphere contacts other than with phosphate oxygens are observed in agreement with experimental results, reproducing that nucleobase nitrogens and carbonyls are weak inner-sphere Mg^2+^-binding sites.^63,75^

In addition, about 20 outer-sphere contacts are formed with *microMg* and *nanoMg*, similar to the results with the Allnér–Villa parameters.^16^ The outer-sphere interactions are mediated by a water molecule and involve besides phosphate oxygens also the oxygen and nitrogen atoms of the nucleobases and the ribose oxygens (Section S2.5).

#### 3.4.2 Identification and de novo Prediction of Inner-Sphere Binding Sites with *NanoMg*

With *nanoMg*, the ion-binding kinetics is 2–4 orders of magnitude faster compared to other force fields. Consequently, the formerly rare events of Mg^2+^ association and dissociation can now be observed directly in straightforward simulations. To evaluate this behavior, in a second step, all ions were placed randomly within the simulation box. Figure 4 shows the distribution of Mg^2+^ ions around the *add* A-riboswitch after a cumulative duration of 2 μs. The riboswitch is surrounded by highly mobile, diffusive ions leading to a low probability density (blue regions). In addition, about 16 Mg^2+^ ions bind in inner-sphere conformation leading to regions of high probability density (red regions). The three ion-binding sites with the highest probability density are shown in Figure 4B–D. Interestingly, the two most probable binding sites correspond to the two inner-sphere binding sites from the X-ray structure. Hereby, the experimental electron density is on top of the probability density calculated from the simulations (Figure 4B,C). This illustrates that the *nanoMg* parameters are particularly useful to predict inner-sphere binding sites. One such prediction, corresponding to the third most likely binding site, is shown in Figure 4D (see Section S2.6 for additional predictions). In addition, the simulations provide an unique atomistic description of the binding site including the exact coordination chemistry of Mg^2+^, RNA, and hydration water.

## 4 Conclusions

The importance of Mg^2+^ in biological systems has driven the development of force field parameters for molecular dynamics simulations. However, Mg^2+^ force fields in combination with TIP3P water have fundamental drawbacks in reproducing a broad range of structural, thermodynamic, and kinetic properties. Our current work shows that the effects of polarizability that are presumably the cause of the deviations can be included by tuning the parameters of the 12–6 Lennard-Jones potential in an enlarged parameter range and by modifying the standard combination rules. Our results show that this allows us to accurately reproduce the experimental solvation free energy, the distances to the oxygens of the first hydration shell, the hydration number, the activity derivative in MgCl_2_ solutions, the self-diffusion coefficient, and the binding affinity and distance to the phosphate oxygens of RNA.

In particular, by increasing the LJ interaction strength between Mg^2+^ and water, the interaction potential becomes more attractive and long-ranged, thereby mimicking the charge-induced dipole and charge-transfer effects that Mg^2+^ ions cause in their environment. This in turn allows us to correctly describe ion–water interactions as quantified by the solvation free energy and the structure of the first hydration shell.

The activity coefficient derivative and the binding affinity to RNA reveal that the Mg^2+^–Cl^–^ and Mg^2+^–RNA interactions described by the standard combination rules are too attractive. The reason is that the combination rules do not reflect the law of matching water affinities according to which ions with high charge density have more tightly bound first hydration shells compared to ions with low charge density.^76^ This problem can easily be solved by introducing scaling factors that reduce the LJ energy of Mg^2+^–Cl^–^ pairs or the Mg^2+^–RNA interactions while simultaneously increasing their effective diameters. The advantage of this approach is that it leaves the ion–water interactions unchanged and can therefore be transferred to other anions or other binding sites on biomolecules. On RNA, for instance, the nucleobase nitrogens and carbonyl atoms could be considered as additional Mg^2+^-binding sites.^62^ However, special care must be taken since the Mg^2+^ ions are unlikely to bind in the inner-sphere binding mode^63,77,78^ and the binding affinity of the inner- and outer-sphere conformation can differ significantly.^69^

In progressing toward improved force fields, special emphasis is placed on an accurate calculation of the water-exchange rate, circumventing the shortcomings of transition state theory.^26^ Matching the abovementioned broad range of thermodynamic properties, we present two sets of optimal parameters: *microMg* which yields water exchange on the microsecond timescale in agreement with experiments and *nanoMg* which yields water exchange on the nanosecond timescale. As shown for the example of the *add* A-riboswitch, *microMg* yields stable RNA structures and reproduces the structure of specifically bound ions. *NanoMg* yields accelerated water exchange and ion-binding dynamics and is therefore particularly suited for the de novo prediction of Mg^2+^-binding sites on biomolecules.

In summary, the Mg^2+^ parameters presented here provide an efficient and highly accurate model for the simulation of Mg^2+^ ions in aqueous solutions and their distribution and exchange around biomolecules such as nucleic acids, proteins, or lipids.

## Supporting Information Available

Additional information regarding the methods presented in the main manuscript are presented. Additional results including a study of box size dependencies on the calculation of solvation free energies, a brief discussion of the performance of Allnér–Villa in combination with mTIP3P water, a study of the dependency of the number of transitions on calculation parameters, a comparison of the two-dimensional free-energy profiles of *microMg* and *nanoMg*, a comparison of different methods to obtain binding affinities and distances, a brief discussion of outer-sphere ion-binding sites, and additional de novo-predicted inner-sphere ion-binding sites are presented (PDF)

## Notes

The parameter files are freely available at https://github.com/bio-phys/Magnesium-FFs.
