# Supplementary material for: Optimized Magnesium Force Field Parameters for Biomolecular Simulations with Accurate Solvation, Ion-Binding, and Water-Exchange Properties
Source: J Chem Theory Comput. 2021 Mar 15;17(4):2530–40. doi: 10.1021/acs.jctc.0c01281 (PMC8047801; doi:10.1021/acs.jctc.0c01281)
Supplement: Supplementary file 1 — ct0c01281_si_001.pdf [file ct0c01281_si_001.pdf]

Supplementary Information: Systematic Optimization of Magnesium Force Field Parameters for Biomolecular Simulations with Accurate Solvation, Ion-pairing, and Water Exchange Properties

Kara K. Grotz, Sergio Cruz-León, and Nadine Schwierz*

Department of Theoretical Biophysics, Max-Planck-Institute of Biophysics, Frankfurt am Main, Germany.

E-mail: nadine.schwierz@biophys.mpg.de

Contents

S1 Supplementary methods **S2**

S1.1 Simulation setup . . . S2

S1.2 Force fields for dimethylphosphate and adenine . . . S2

S1.3 Solvation free energy calculation . . . S4

S1.4 Single-ion properties R_1, n_1, and D_0 . . . S5

S1.5 Calculation of free energy profiles . . . S6

S1.6 Transition state theory for k_{TST} . . . S7

S1.7 Counting transitions . . . S8

S1.8 Activity derivatives . . . S9

S1.9 Computation of binding affinities . . . S10

S2 Supplementary results **S12**

S2.1 Comparison of Lennard-Jones parameters . . . S12

S2.2 Allnér-Villa with mTIP3P . . . S13

S2.3 Two dimensional free energy landscapes . . . S15

S2.4 $\Delta G_{\mathrm{b}}^{0}$ and R_{b} calculation and comparison of different methods . . . S15

S2.5 Outer-sphere ion binding sites with *microMg* and *nanoMg* S18
S2.6 Identification and de novo prediction of inner-sphere binding sites with *nanoMg* S18
S3 Bibliography **S20**

S1 Supplementary methods

Throughout this work, multiple different methods have been applied. Here, we provide additional information regarding the methods presented in the main manuscript.

S1.1 Simulation setup

In all simulations, energy minimization by steepest decent was followed by NVT and NPT equilibration simulations of 0.5 ns and 1 ns, respectively. The temperature was kept at 300 K and the pressure at 1 atm using the Berendsen thermostat and barostat,[1] respectively. During equilibration, we applied position restraints to all ions to avoid ion-paring before hydration shells could form properly. For all production runs, P-LINCS[2] was used to constrain bonds involving hydrogen atoms to their equilibrium lengths, enabling a time step of 2 fs. Real-space van der Waals and electrostatic interactions were truncated at 1.2 nm. Long-range electrostatics were computed using the particle mesh Ewald method[3] with a Fourier spacing of 0.12 nm. Using the velocity-rescale thermostat by Bussi et al.[4] and the Parrinello-Rahman barostat,[5] the simulations were performed at 300 K and 1 atm. Trajectories for the 12-6-4 force field by Li-Merz[6] were obtained with AMBER[7] subsequently converted into GROMACS[2] format. Multiple systems were simulated during this study and are listed in Table S1.

S1.2 Force fields for dimethylphosphate and adenine

For small compounds lacking a specialized force field, one the most adequate models to resemble and interact with currently used AMBER-derived force fields is the general amber force field (GAFF).[8] The dimethylphosphate (DMP) and the adenine ligand of the *add* A-riboswitch were hence de novo parametrized using the automated atom type and bond type perception of Antechamber[9] to obtain a GAFF force field.[10,11] RESP partial charges were obtained at HF/6-31G* level of theory in vacuum. The charges on atoms belonging to the phosphate group of the DMP were slightly adjusted to match those of the phosphate group in the parmBSC0χ_{OL3} force field.[12–14] The remaining partial charges on the two methyl groups were subsequently adjusted slightly to be symmetric and to give an integer net charge (Table S2). As the DMP was used to mimic the phosphate group of RNA and because in this work the Mg^{2+} force fields are optimized to be

Table S1: Throughout this work, various systems were simulated, that are described here in more detail. 'Phys. property' names the physical properties that were obtained from the respective simulation. 'Method' describes the kind of simulation that was performed to obtain the desired quantity, with FEP being free energy perturbation calculations, unbiased being straight forward simulations without any additional bias, und US being Umbrella Sampling simulations. In 'System' all particles of the respective simulation are listed (with DMP: dimethylphosphate). 'Duration' lists the duration of the respective simulations, with products indicating for FEP and US simulation the various simulation windows. Lastly, 'L' lists the edge lengths of the simulation boxes, where (cu) indicates cubic box and (do) a rhombic dodecahedron.

Phys. property	Method	System	Duration	L
ΔG_{solv}, R_1, n_1	FEP	1 Mg^{2+}, 506 water	40 × 1 ns	2.5 nm (cu)
D_0	unbiased	1 Mg^{2+}, 506 water	100 ns	2.5 nm (cu)
a_{cc}, k	unbiased	39 Mg^{2+}, 40 Cl^{-}, 2048 water (1 M)	1 μs	4 nm (cu)
a_{cc}	unbiased	73 Mg^{2+}, 146 Cl^{-}, 1961 water (2 M)	150 ns	4 nm (cu)
a_{cc}	unbiased	40 Mg^{2+}, 20 Cl^{-}, 2105 water (0.5 M)	150 ns	4 nm (cu)
a_{cc}	unbiased	20 Mg^{2+}, 10 Cl^{-}, 2135 water (0.25 M)	150 ns	4 nm (cu)
k_{TST}, $F(r_{\mathrm{MgOw}})$	US	1 Mg^{2+}, 505 water	86 × 3 ns	2.5 nm (cu)
$F(r_{\mathrm{MgOw_1}}, r_{\mathrm{MgOw_2}})$	US	1 Mg^{2+}, 506 water	946 × 5 ns	2.5 nm (cu)
ΔG^0_{b}	FEP	1 DMP, 1 Mg^{2+}, 3 Na^{+}, 4 Cl^{-}, 1470 water (0.1 M)	20 × 3 ns	4 nm (do)
ΔG^0_{b}, $F(r_{\mathrm{MgOP}})$	US	1 DMP, 1 Mg^{2+}, 3 Na^{+}, 4 Cl^{-}, 1470 water (0.1 M)	67 × 20 ns	4 nm (do)
$\Delta G^0_{\mathrm{Ca^{2+}}}$, $F(r_{\mathrm{CaOP}})$	US	1 DMP, 1 Ca^{2+}, 3 Na^{+}, 4 Cl^{-}, 1470 water (0.1 M)	67 × 20 ns	4 nm (do)
Evaluation of performance	unbiased	1 *add* A-riboswitch (PDB ID:1y26, incl. adenine), 35 Mg^{2+}, 30,664 water	100 ns, 500 ns, 10 × 200 ns	11.1 nm (do)

Table S2: Partial charges used for the dimethylphosphate (DMP). The DMP was de novo parametrized using the automated atom type and bond type perception of Antechamber[9] to obtain a GAFF force field.[8,10,11]

atom type	residue name	atom name	partial charge
c3	DMP	C1	0.1220
h1	DMP	H11	0.0166
h1	DMP	H12	0.0166
h1	DMP	H13	0.0166
os	DMP	O2	-0.4789
p5	DMP	P3	1.1662
o	DMP	O4	-0.7760
o	DMP	O5	-0.7760
os	DMP	O6	-0.4789
c3	DMP	C7	0.1220
h1	DMP	H71	0.0166
h1	DMP	H72	0.0166
h1	DMP	H73	0.0166

used with RNA as described by parmBSC0χ_{OL3},[12–14] we furthermore employed the angle definition of the parmBSC0χ_{OL3} force field for $V_a(\theta_{ijk})$ between the atoms O1P-P-O2P (Table S3). The harmonic angular potential $V_a(\theta_{ijk})$ of the angle θ_{ijk} between the three atoms i, j, and k is defined by

$$V_a(\theta_{ijk}) = \frac{1}{2}k^{\theta}_{ijk}(\theta_{ijk} - \theta^0_{ijk})^2, \tag{S1}$$

with force constant k^{θ}_{ijk} and equilibrium angle θ^0_{ijk}.

Table S3: Definition of the angle between non-bridging phosphate oxygens and the phosphorous atom of the DMP obtained from the GAFF approach and as defined by the parmBSC0χ_{OL3} force field. The angle potential is defined by eq S1. In this work, the angle definition according to OL3 was used.

	i	j	k	θ^0_{ijk}	k^{θ}_{ijk}
GAFF	O4	P	O5	115.8	383.25
OL3	O1P	P	O2P	119.9	1171.52

S1.3 Solvation free energy calculation

We calculated the solvation free energy in a parameter range of σ_{io} = 0.16 - 0.24 nm and ϵ_{io} = 1.8 - 28 kJ/mol with free energy perturbation (FEP) simulations and the Bennet acceptance ratio (BAR) method.[15] The statistical uncertainty of the method is around 1 kJ/mol. The single ion was solvated with 506 water molecules in a cubic box with edge length of 2.5 nm. The free energy perturbation was performed over 40 evenly spaced replicas, where in the first half of the replicas a neutral LJ particle was created and in the second half the charge increased until it reached a net charge of +2. Each of these replicas was 1 ns long. The first 200 ps of each replica were excluded from the analysis to extend the equilibration phase.

To be able to compare to experimental measurements, several corrections to the raw simulation data are required. To account for finite size effects, the correction term is the following[16]

$$\Delta G_{\mathrm{fs}} = \frac{z^2 N_A e^2}{4\pi\epsilon_0}\left[-\frac{\zeta_{\mathrm{ew}}}{2\epsilon_r} + \left(1 - \frac{1}{\epsilon_r}\right)\left(\frac{2\pi R_1^2}{3L^3} - \frac{8\pi^2 R_1^5}{45L^6}\right)\right] \tag{S2}$$

for an ion with valency z. N_A is Avogardo's number, e the unit charge, ϵ_0 the vacuum permittivity, R_1 the first peak of the ion-water radial distribution function and hence the effective ion radius, and ϵ_r = 83 the relative dielectric constant of TIP3P water.[17] The Wigner potential is given as $\zeta_{\mathrm{ew}} = -2.837279/L$ with L being the edge length of the simulation box in nm.[18,19] Additional terms[20,21] include corrections related to the compression of ideal gas vs. ideal solution $\Delta G_{\mathrm{press}}$ = 7.9 kJ/mol and the experimental transfer of ions through the ion-water interface $\Delta G_{\mathrm{surf}} = -z \times 50.8$ kJ/mol.[22] In total, the solvation free energy of a single

ion X is given by

$$\Delta G^X_{\mathrm{solv}} = \Delta G^X_{\mathrm{sim}} + \Delta G_{\mathrm{fs}} + \Delta G_{\mathrm{surf}} + \Delta G_{\mathrm{press}} \ . \tag{S3}$$

We use experimental solvation free energies for a neutral $MgCl_2$ pair ΔG_{solv} with cation-valency $z = 2$ in our force field optimization and employ the solvation free energy of Cl^- $\Delta G^{\mathrm{Cl}}_{\mathrm{solv}} = 303.9$ kJ/mol obtained in our previous work[21] (eq S4) as for ion-pairs the experimental data is more robust then for single ions,[20,21]

$$\Delta G_{\mathrm{solv}} = \Delta G^{\mathrm{Mg}}_{\mathrm{solv}} + z \times \Delta G^{\mathrm{Cl}}_{\mathrm{solv}} \ . \tag{S4}$$

To test the influence of the system size, three different box sizes were used (2.5 nm, 3.5 nm, and 4.5 nm). As shown in Table S4, no dependence on the size of the simulation box could be observed in agreement with previous findings.[19,20]

Table S4: Calculated solvation free energies ΔG_{solv} for the final parameter sets *microMg* and *nanoMg* using simulation boxes of different sizes. Note that ΔG_{solv} takes the corrections for finite size, interfacial crossing, and pressure into account via eq S2-S4.

	box edge length [nm]	ΔG_{solv} [kJ/mol]
microMg	2.5	-2532.9 ± 1
	3.5	-2533.1 ± 1
	4.5	-2533.1 ± 1
nanoMg	2.5	-2532.0 ± 1
	3.5	-2532.0 ± 1
	4.5	-2532.9 ± 1
exp.		-2532

S1.4 Single-ion properties R_1, n_1, and D_0

The average distance between the Mg^{2+} ion and the water of its first hydration shell R_1 is determined by evaluating the radial distribution function (rdf) $g(r)$ of magnesium and the water oxygens. The first peak of the rdf corresponds to the first hydration shell around the cation. Integrating the area under the first peak of $g(r)$ hence gives rise to the coordination number n_1 of water molecules in this first hydration shell,

$$n_1 = 4\pi\rho \int_0^{\mathrm{r_{min}}} g(r) r^2 dr \ , \tag{S5}$$

with the bulk water density ρ. r_{min} is the minimum of the rdf after the first peak.

For *microMg* and *nanoMg*, diffusion coefficients were calculated from additional 100 ns long NVT simulations. The first 10 ns were excluded from the analysis. The coefficients $D_{\mathrm{pbc}}(L)$ were calculated from a straight line fit of the slope of the mean-squared displacement of the single ion, taking only the linear part

into account. The obtained diffusion coefficient was size-corrected[23] by

$$D_0 = \frac{\eta_{\text{TIP3P}}}{\eta_{\text{water}}}\left[D_{\text{pbc}}(L) + \frac{k_B T \zeta_{\text{ew}} \alpha}{6\pi\eta L}\right], \tag{S6}$$

with L being the edge length of the simulation box, k_B the Boltzmann constant, T the absolute temperature, η the solvent viscosity, and $\zeta_{\text{ew}} = 2.837297$ the self-term for a cubic lattice. We used the empirical parameter $\alpha = 1$. Moreover, to correct for the low viscosity of TIP3P water, we scaled the diffusion coefficient with the viscosity of TIP3P water $\eta_{\text{TIP3P}} = 3.13 \times 10^{-4}$ kg m^{-1} s^{-1} and the experimentally determined viscosity of water $\eta_{\text{water}} = 8.91 \times 10^{-4}$ kg m^{-1} s^{-1}.[24] Errors in the self-diffusion coefficient correspond to the standard error of the mean from dividing each trajectory into 3 blocks and block averaging.[25]

S1.5 Calculation of free energy profiles

In this work, all free energy profiles are obtained by Umbrella Sampling.[26,27] Subsequently, in all cases the weighted histogram analysis method (WHAM)[28] is used to combine the individual umbrella windows to a free energy profile $F(r)$.

1D Mg^{2+} - water: The one dimensional free energy profiles as a function of the distance between Mg^{2+} and the oxygen atom of the leaving water molecule employing 12-6 type force fields were obtained with GROMACS[2] using force constants and window spacing of $k = 400,000$ kJ/(mol nm^2) and 0.005 nm [r_{MgOw_1} = 0.17 - 0.4 nm], $k = 100,000$ kJ/(mol nm^2) and 0.01 nm [r_{MgOw_1} = 0.4 - 0.6 nm] and $k = 1,000$ kJ/(mol nm^2) and 0.02 nm [r_{MgOw_1} = 0.6 - 1 nm], respectively. The same force constants and window spacing values were used for the one dimensional profile employing the 12-6-4 type force field but here in combination with the simulation engine AMBER[7] (version 2020) and PLUMED[29] (version 2.5). All windows were simulated for 3 *ns* each.

2D Mg^{2+} - water: For *microMg* and *nanoMg* furthermore two dimensional free energy profiles were obtained including also the distance towards the oxygen of the incoming water molecule r_{MgOw_2} and not only towards the outgoing one r_{MgOw_1}. Here, a force constant of $k = 100,000$ kJ/(mol nm^2) and a window spacing of 0.01 nm were used along the distances. The profiles were obtained with GROMACS[2] and PLUMED.[29] Additionally, a second type of restraint was employed acting on all water oxygens using a switching function (eq S7) to calculate the coordination number

$$s_{\text{ij}} = \frac{1 - \left(\frac{\boldsymbol{r}_{\text{ij}}}{r_0}\right)^n}{1 - \left(\frac{\boldsymbol{r}_{\text{ij}}}{r_0}\right)^m}\ , \tag{S7}$$

with $r_0 = 0.3\ nm$, $n = 24$, and $m = 288$. To force the non-exchanging water molecules to remain within the first hydration shell while forcing all other waters to remain outside, the coordination number was restrained to five or zero, respectively. The idea behind this procedure is that all $N-5$ states are identical and it is thus sufficient to sample only exchanges between two selected states.[30] A force constant of $k = 1,000$ kJ/(mol nm^2) was used, the windows were simulated for 5 ns each.

1D Mg^{2+} - phosphate oxygen: A third type of free energy profile was calculated along the distance between Mg^{2+} and one of the two non-bridging phosphate oxygens of the dimethylphosphate (DMP) using force constants and window spacing of $k = 300,000$ kJ/(mol nm^2) and 0.0075 nm [$r_{\mathrm{MgOP}} = 0.15$ - 0.525 nm] and $k = 5,000$ kJ/(mol nm^2) and 0.02 nm [$r_{\mathrm{MgOP}} = 0.525$ - 0.885 nm], respectively. We calculated the free energy profiles using PLUMED[29] including an additional bias to avoid any direct interaction of the Mg^{2+} ion with any atom of the DMP but the respective phosphate oxygen. Similar to the hydration restraints in the 2D Mg^{2+} - water profiles also here a switching function (eq S7) is used to bias the direct interaction. This additional bias is required to ensure that we sample only transitions between inner-shell binding to the selected phosphate oxygen and an outer-shell configuration. The windows were 20 ns long each. Convergence was ensured by dividing the windows in four blocks of 5 ns each.

1D Ca^{2+} - phosphate oxygen: The free energy profiles between Ca^{2+} and DMP were obtained exactly as the Mg^{2+} - DMP free energy profiles as described in the paragraph above. We calculated two free energy profiles using PLUMED.[29] One was obtained without any further bias and a second one including the additional bias to avoid any direct interaction of the Ca^{2+} ion with any atom of the DMP but the respective phosphate oxygen. The parameters for Ca^{2+} are taken from Mamatkulov-Schwierz.[21]

S1.6 Transition state theory for k_{TST}

The potential of mean force V^{PMF} used for the rate calculation using Transition State Theory (k_{TST}) is obtained from the free energy profile along the distance between Mg^{2+} and the oxygen atom of the leaving water molecule $r_{\mathrm{MgOw_1}}$ (here $r_{\mathrm{MgOw_1}} = r$ for clarity). The free energy profile is calculated (section S1.5) and afterwards a Jacobian correction is applied

$$V^{\mathrm{PMF}}(r) = F(r) + 2k_B T \ln r, \tag{S8}$$

to take into account that we use radial distances rather the Cartesian coordinates.[31] From this V^{PMF} the rate coefficient k_{TST} is calculated via,

$$k_{\mathrm{TST}} = \sqrt{\frac{k_B T}{2\pi\mu}} \cdot \frac{r^{*2} e^{-\beta \mathrm{V}^{\mathrm{PMF}}(\mathrm{r}^*)}}{\int_0^{\mathrm{r}^*} r^2 e^{-\beta \mathrm{V}^{\mathrm{PMF}}(\mathrm{r})} dr} \ , \tag{S9}$$

where k_B is Boltzmann's constant, μ is the reduced mass of the Mg^{2+}-H_2O pair and r^* the distance of the top of the barrier of the potential of mean force V^{PMF}.

S1.7 Counting transitions

To calculate the exchange rate coefficient k, we produced 1 μs long trajectories at 1 M $MgCl_2$. Each water molecule was followed individually throughout the trajectory. We calculated the distance of its oxygen atom to any Mg^{2+} in the simulation box for each frame of the trajectory. This distance was converted into an indicator function $h(r)$ being 1 if the molecule is part of the first hydration shell of any Mg^{2+} and 0 otherwise,

$$h(r) = \left\{ \begin{array}{ll} 0 & \text{for } r > r_{\mathrm{ub}} \\ (1 + e^{\beta(\mathrm{r} - \lambda \mathrm{r_b})})^{-1} & \text{for } r_{\mathrm{ub}} > r > r_{\mathrm{b}} \\ 1 & \text{for } r < r_{\mathrm{b}} \end{array} \right\} \tag{S10}$$

with $r_{\mathrm{b}} = 0.28$ nm, $r_{\mathrm{ub}} = 0.35$ nm, $\lambda = 1.05$, $\beta = 5$ to avoid counting of fast recrossings. Only distances crossing both r_{b} and r_{ub} and staying in the new state for at least 4 ps (two frames) were considered as transitions. The values for r_{b} and r_{ub} were chosen from the free energy profile with r_{b} being close to the top of the barrier and r_{ub} in between the barrier top and the second minimum in the free energy profile (Figure S1). To test the robustness of the parameters, we have tested a broad range of possible vales for r_{b} and r_{ub}. The results show that the calculated rate does not change significantly if the definition of the parameters is varied (Table S5).

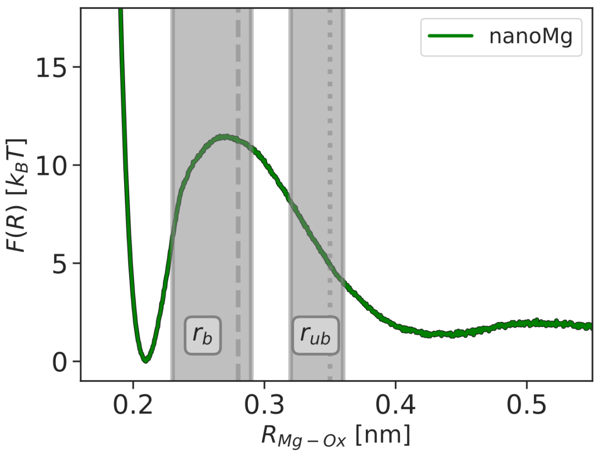

Figure S1: One dimensional free energy profiles for *nanoMg* (green) and definition of the cutoff parameters r_{b} (dashed) and r_{ub} (dotted) that define the stable states. The gray area indicate the parameter range tested (Table S5).

Table S5: Number of water exchanges N from the first hydration shell of Mg^{2+} in a 1 μs trajectory of 1 M $MgCl_2$ for both *microMg* and *nanoMg* with respect to varying cutoff parameters r_{b} and r_{ub} of the indication function $h(r)$ (eq S10).

	r_{b} [nm]	r_{ub} [nm]	N (*microMg*)	k (*microMg*) [s^{-1}]	N (*nanoMg*)	k (*nanoMg*) [s^{-1}]
This study	0.28	0.35	376 ± 56	$(8.04 \pm 1.20) \cdot 10^5$	52086 ± 120	$(1.11 \pm 0.003) \cdot 10^8$
set2	0.28	0.36	380 ± 56	$(8.12 \pm 1.20) \cdot 10^5$	52232 ± 124	$(1.12 \pm 0.003) \cdot 10^8$
set3	0.29	0.35	386 ± 58	$(8.25 \pm 1.24) \cdot 10^5$	52180 ± 130	$(1.12 \pm 0.003) \cdot 10^8$
set4	0.27	0.35	374 ± 54	$(7.99 \pm 1.15) \cdot 10^5$	52018 ± 140	$(1.11 \pm 0.003) \cdot 10^8$
set5	0.28	0.34	376 ± 56	$(8.04 \pm 1.20) \cdot 10^5$	51988 ± 110	$(1.11 \pm 0.003) \cdot 10^8$
set6	0.29	0.36	406 ± 46	$(8.68 \pm 0.98) \cdot 10^5$	52380 ± 132	$(1.12 \pm 0.003) \cdot 10^8$
set7	0.27	0.34	376 ± 54	$(7.99 \pm 1.15) \cdot 10^5$	51934 ± 124	$(1.11 \pm 0.003) \cdot 10^8$
set8	0.23	0.32	372 ± 52	$(7.95 \pm 1.11) \cdot 10^5$	51122 ± 56	$(1.09 \pm 0.001) \cdot 10^8$

S1.8 Activity derivatives

The activity coefficient a_c and hence its derivative a_{cc} give the experimentally measured deviation from ideal behavior. For non-interacting particles, *i.e.*, ideal solutions, the activity coefficient is 1. Hence, any interaction between particles is expressed as a deviation from unity. Capturing this deviation with MD simulations provides a force field with proper balance between both ion-ion interactions as well as water-ion interactions. We use Kirkwood-Buff (KB) theory[32] to compute the activity derivative of the $MgCl_2$ electrolyte solution at various concentrations and to be able to compare it to the abundant experimental data for a_{cc}. This makes it comparably simpler to account for the ion-ion interaction in MD simulations than calculating directly the activity coefficient. Using pair distribution function between two species i and

j, the so-called KB integrals G_{ij}, KB theory grants expressions for a variety of thermodynamic properties,

$$G_{ij} = 4\pi \int_0^\infty \left[g_{ij}^{\mu\mathrm{VT}}(r_{ij}) - 1\right] r_{ij}^2 dr_{ij} \ , \tag{S11}$$

where $g_{ij}^{\mu\mathrm{VT}}(r_{ij})$ is the radial distribution function of the two species in the grand canonical ensemble, with r_{ij} being the center of mass distance between the two. The KB integrals are defined for infinite space and open systems (μVT). MD simulations are in most cases performed in closed systems (NVT, NPT) and due to the finite size of simulation boxes the integrals cannot be solved for infinite space. Hence, KB integrals are commonly truncated after a distance R,

$$G_{ij}^{\mathrm{rescale}} = 4\pi \int_0^R \left[g_{ij}^{\mathrm{NPT}}(r^{'}\rho) - 1\right] r^2 dr \ , \tag{S12}$$

where we rescale the rdf via $g_{ij}(r_{ij}^{'}\rho) = f(\rho) g_{ij}^{\mathrm{sim}}(r_{ij})$ to ensure unity at large distances.[21,33]

The activity derivate a_{cc} is defined as

$$a_{cc} = \left(\frac{\partial \ln a_c}{\partial \ln \rho_c}\right)_{\mathrm{P,T}} = 1 - \left(\frac{\partial \ln y_c}{\partial \ln \rho_c}\right)_{\mathrm{P,T}} = \frac{1}{1 + \rho_c(G_{\mathrm{cc}} - G_{\mathrm{co}})} \ , \tag{S13}$$

with the activity $a_c = \rho_c y_c$, the cosolvent molar activity coefficient y_c and number density ρ_c. For divalent cations the required expressions[33,34] are

$$G_{\mathrm{cc}} = \frac{1}{9}\Big[G_{++} + 4(G_{--} + G_{+-})\Big] \tag{S14}$$

and

$$G_{\mathrm{co}} = G_{\mathrm{oc}} = \frac{1}{3}G_{+\mathrm{o}} + \frac{2}{3}G_{-\mathrm{o}} \ , \tag{S15}$$

where $+$, $-$, and o denote cation, anion, and water oxygen, respectively.

S1.9 Computation of binding affinities

Alchemical Transformation: Multiple scaling factor combinations $\lambda_{\sigma,\epsilon}^{\mathrm{RNA}}$ were screened by calculating the binding affinities towards the phosphate oxygens of the dimethylphosphate using alchemical transformation calculations

$$\Delta G_{\mathrm{b}}^0 = \Delta G_{\mathrm{Mg^{2+}\to Ca^{2+}}}^{\mathrm{solv}} + \Delta G_{\mathrm{Ca^{2+}}}^0 + \Delta G_{\mathrm{Ca^{2+}\to Mg^{2+}}}^{\mathrm{bind}} \ , \tag{S16}$$

where the binding affinity of Ca^{2+} ($\Delta G_{\mathrm{Ca^{2+}}}^0$) was used as reference.[35] The indices $Mg^{2+} \to Ca^{2+}$ and $Ca^{2+} \to Mg^{2+}$ denote the alchemical transformation from Mg^{2+} to Ca^{2+} in the bulk state and from Ca^{2+} to Mg^{2+}

in the bound state, respectively. Additionally, the distance between Mg^{2+} and the phosphate oxygen it is bound to R_b was obtained by averaging over this distance over 100 ns long unbiased trajectories.

Integration of V^PMF: Binding affinities for the interaction between Mg^{2+} and the phosphate oxygen were furthermore obtained by integration of the PMF along the distance between them r_MgOP (here $r = r_\mathrm{MgOP}$ for clarity),

$$\Delta G_\mathrm{b}^0 = -k_B T \cdot \ln\left(\frac{c^0}{[M]} \cdot \frac{\int_0^{\mathrm{r}^\dagger} r^2 e^{-\mathrm{V}^\mathrm{PMF}(\mathrm{r})/\mathrm{k_B T}} dr}{\int_{\mathrm{r}^\dagger}^{\mathrm{r^L}} r^2 e^{-\mathrm{V}^\mathrm{PMF}(\mathrm{r})/\mathrm{k_B T}} dr}\right) , \tag{S17}$$

with the standard concentration $c^0 = 1$ M, the concentration of Mg^{2+} in the simulation box $[M] = 0.025M$, the position of the maximum of the PMF $r^\dagger$, the radius of a sphere that contains the same number of water molecules as our simulation box r_L. Errors are calculated by dividing the 20 ns long windows into 5 blocks, and subsequent calculation of PMFs and thus ΔG_b^0 from each block and block averaging.

S2 Supplementary results

In the following section, we present additional results to the main text.

S2.1 Comparison of Lennard-Jones parameters

For comparison, the optimized Lennard-Jones (LJ) parameter combinations of *microMg* and *nanoMg* are listed together with parameter combinations for Mg^{2+} from the literature[6,21,36,37] (Table S6). These parameter combinations as well as the isoareas for the solvation free energy of the neutral ion pair ΔG_{solv}, the average radius of the first hydration shell R_1, and the coordination number of the first hydration shell n_1 as obtained from the grid search (see section S1.3, S1.4) are shown in Figure S2.

Table S6: Optimized force field parameters for Mg^{2+} for simulations with the TIP3P[38] water model. σ_{ii} and ε_{ii} are the ion-ion, σ_{io} and ε_{io} the ion-water Lennard-Jones parameter, respectively (eq 1,2, main text). In comparison σ and ε values for Mg^{2+} force fields from the literature[6,21,36,37] are shown.

units	σ_{ii} [nm]	ε_{ii} [kJ/mol]	σ_{io} [nm]	ε_{io} [kJ/mol]	κ [Å^{-2}]
microMg	0.101934	235.804	0.2085	12.25	n.a.
nanoMg	0.102534	389.799	0.2088	15.75	n.a.
Allnér-Villa[36]	0.276939	0.012363	0.2960	0.0887	n.a.
Mamatkulov-Schwierz[21]	0.161939	0.604036	0.2385	0.6200	n.a.
Li-Merz[37] (12-6)	0.228739	0.016573	0.2719	0.1027	n.a.
Li-Merz[6] (12-6-4)	0.256044	0.094473	0.2452	0.2856	1.046

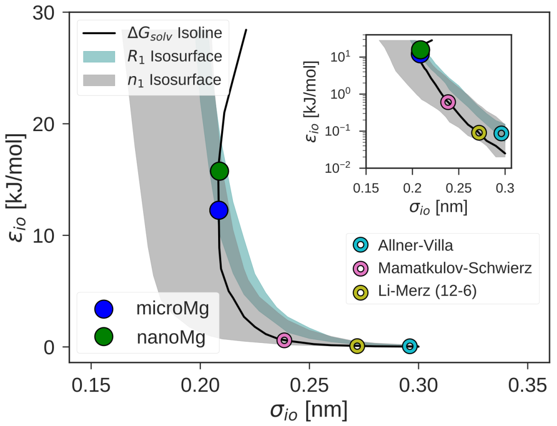

Figure S2: Single-ion property isosurfaces in σ_{io} - ε_{io} space for the interaction of *microMg*, *nanoMg* and force fields from the literature[21,36,37] in interaction with TIP3P water.[38]

S2.2 Allnér-Villa with mTIP3P

Ion force fields are typically developed in combination with a specific water model. There is no guaranty that physical properties that have been reproduced in the optimization procedure in combination with one water model will still be reproduced using the respective ion force field in combination with a different water model. In this work, Mg^{2+} force fields as used in combination with the TIP3P water model[38] are discussed. Because frequently used, we also included here the Allnér-Villa force field.[36] This force field, however, has been developed using CHARMM,[39] where not the TIP3P water model is employed but a slightly modified version, the mTIP3P.[40] Both TIP3P and mTIP3P carry the same partial charges on both oxygen and hydrogens, respectively, as well as the same σ and ε parameters for the LJ interaction of the oxygen atom with its surroundings. However, on top of that, the mTIP3P additionally contains LJ parameters for the description of the interaction of the hydrogens. This small and often overlooked difference has severe consequences, as, *e.g.*, the solvation free energy ΔG_{solv} of the Allnér-Villa(mTIP3P) force field differs by more than 100 kJ/mol from that of Allnér-Villa(TIP3P) (Figure S3A). While for Allnér-Villa(mTIP3P) ΔG_{solv} is closer to the experimental value, for Allnér-Villa(TIP3P) there is quite a discrepancy. Comparing the free energy profiles of Allnér-Villa(mTIP3P) and Allnér-Villa(TIP3P) (Figure S3C), there is a clear difference of 1.5 $k_B T$ at the top of the barrier. Hence, not only in terms of thermodynamics there is a difference between using TIP3P and mTIP3P, also the kinetics change. Interestingly, this change in the kinetics is not large enough to change the number of transitions significantly that could be counted within a 1 μs trajectory (Figure S3B, Table S7).

Table S7: Number of counted water exchanges from the first hydration shell of Mg^{2+} in a 1 μs trajectory of 1 M $MgCl_2$, computed water exchange rate coefficients from counting k (eq 5, main text), and rate coefficients as calculated from transition state theory k_{TST} for *microMg*, *nanoMg* and Mg^{2+} force fields from the literature.

units	N	k [s^{-1}]	k_{TST} [s^{-1}]
microMg	376 ± 56	(8.04 ± 1.20) $\cdot 10^5$	2.25 $\cdot 10^6$
nanoMg	52086 ± 120	(1.11 ± 0.003) $\cdot 10^8$	2.01 $\cdot 10^8$
Mamatkulov-Schwierz[21]	2 ± 2	n.a.	2.94 $\cdot 10^4$
Allnér-Villa[36] [TIP3P]	2 ± 2	n.a.	2.39 $\cdot 10^5$
Allnér-Villa[36] [mTIP3P]	24 ± 12	n.a.	2.03 $\cdot 10^6$
Li-Merz[37] (12-6)	2 ± 2	n.a.	3.52 $\cdot 10^4$
Li-Merz[6] (12-6-4)	6720 ± 160	(1.44 ± 0.03) $\cdot 10^7$	9.54 $\cdot 10^7$
exp.[41,42]	248 - 314	$5.3 - 6.7 \cdot 10^5$	n.a.

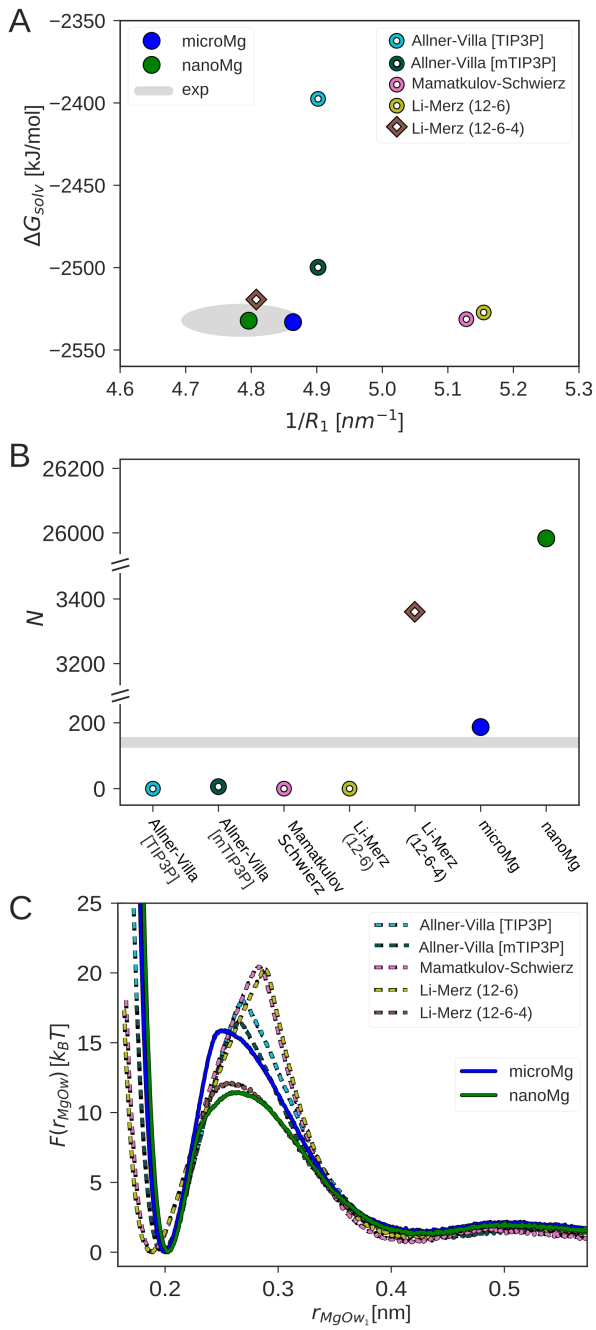

Figure S3: (A) Solvation free energy of neutral $MgCl_2$ pairs $\Delta G_{\rm solv}$ calculated for different Mg^{2+} force fields. For the ion pairs described by Allnér-Villa,[36] Li-Merz (12-6),[37] and Li-Merz (12-6-4)[6] $\Delta G^{\rm Cl}_{\rm solv}$ = - 347 kJ/mol from Marcus,[43] for Mamatkulov-Schwierz,[21] *microMg*, and *microMg* $\Delta G^{\rm Cl}_{\rm solv}$ = - 303.9 kJ/mol from ref[21] after Tissandier.[44] (B) Number of exchange events as observed in a 1 μs long 1 M $MgCl_2$ simulation. The experimental count is obtained from refs.[41,42] and eq 5, main text. (C) One dimensional free energy profiles $F(r_{\rm MgOw})$ along the distance $r_{\rm MgOw}$ between Mg^{2+} and the oxygen of the leaving water molecule for different Mg^{2+} force fields.

S2.3 Two dimensional free energy landscapes

Since the one dimensional representation is insufficient to capture the exchange mechanism of water exchange,[30] we have calculated two dimensional free energy profiles that take into account the coordinates of the incoming r_{MgOw_2} and leaving r_{MgOw_1} water molecules (Figure S4).

From the differences in rate calculations and one dimensional free energy profiles (Figure S3B,C and Figure 2, main text) we expect also the two dimensional free energy profiles to differ between *microMg* and *nanoMg*. The transition from the upper left corner of Figure S4A or B to its lower right corner (that corresponds to an exchange between incoming and outgoing water; compare exchange pathways in Figure 2C, main text) leads for *microMg* through an area that is higher in energy than the corresponding one for *nanoMg* (lower left corners of Figure S4A,B).

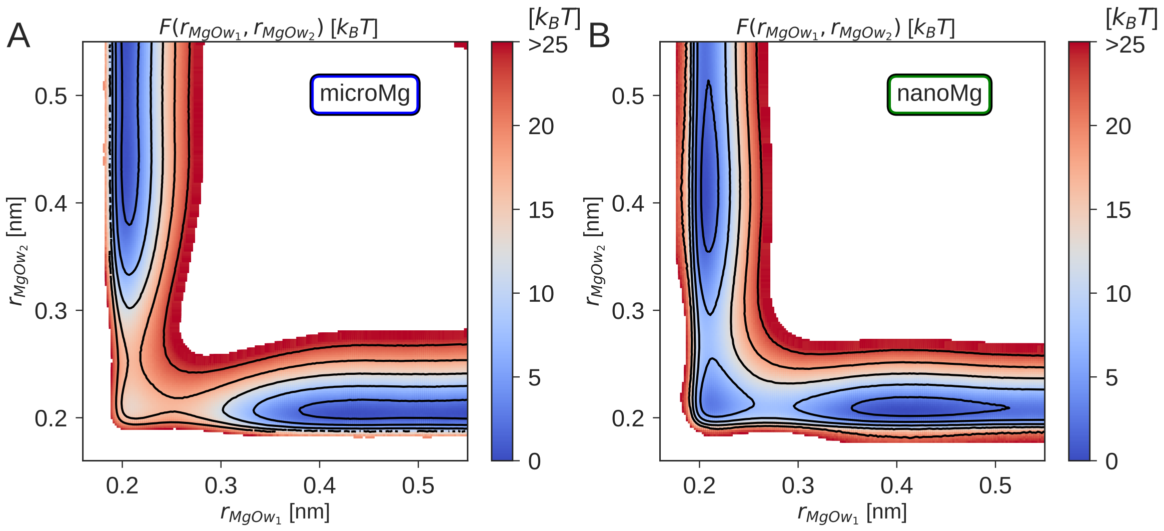

Figure S4: Two dimensional free energy profiles $F(r_{\mathrm{MgOw}_1}, r_{\mathrm{MgOw}_2})$ along the distances between Mg^{2+} and the oxygens of the incoming r_{MgOw_2} and leaving r_{MgOw_1} water molecules for (A) *microMg* and (B) *nanoMg*. The energy contour spacing is 5 k_BT.

S2.4 ΔG_{b}^0 and R_{b} calculation and comparison of different methods

Two different methods, namely alchemical transformation and integration of free energy profiles, were applied to calculate the binding affinity ΔG_{b}^0 of the dimethylphosphate (DMP).

Alchemical transformation: In a first step, the binding affinity of Ca^{2+}, $\Delta G^0_{\mathrm{Ca}^{2+}} = -4.842 \pm 0.182$ $\mathrm{k_BT}$, and the change in solvation free energy upon transformation of Mg^{2+} into Ca^{2+}, $\Delta G^{\mathrm{solv}}_{\mathrm{Mg}^{2+}\rightarrow\mathrm{Ca}^{2+}}$, were obtained. The former was done by calculating the one dimensional free energy profile along the Ca^{2+} phosphate distance and its subsequent integration (see next paragraph). The latter was calculated using

free energy perturbation calculation. As these two quantities are independent of the interaction between Mg^{2+} and DMP, they also remain constant while applying different scaling factors $\lambda_{\sigma,\varepsilon}^{\mathrm{RNA}}$ and hence had only to be calculated once. Only the last step to close the alchemical circle (eq S16), $\Delta G^{\mathrm{bind}}_{\mathrm{Ca}^{2+}\rightarrow\mathrm{Mg}^{2+}}$, was left to calculate on a grid of $\lambda_{\sigma,\varepsilon}^{\mathrm{RNA}}$ values. This made the calculation of the binding affinity using alchemical transformation quite efficient. To ensure convergence, the last step was calculated both in forward (as in eq S16, Table S8) as well as backward direction (Table S9). The scaling factor combinations resembling the experimental values closest were selected from a range of $\lambda_{\sigma}^{\mathrm{RNA}}$ = 0.97 - 1.23 and $\lambda_{\varepsilon}^{\mathrm{RNA}}$ = 0.08 - 1.04. R_{b} results from averaging over the Mg^{2+}-OP distance during 100 ns long straight forward simulation.

Table S8: Binding affinity $\Delta G_{\mathrm{b}}^{0}$ obtained from forward alchemical transformation. The values given for $\Delta G^{\mathrm{solv}}_{\mathrm{Mg}^{2+}\rightarrow\mathrm{Ca}^{2+}}$ and $\Delta G^{\mathrm{bind}}_{\mathrm{Ca}^{2+}\rightarrow\mathrm{Mg}^{2+}}$ are obtained from block averaging for three blocks of 3 ns long windows each. $\Delta G^{\mathrm{bind}}_{\mathrm{Ca}^{2+}\rightarrow\mathrm{Mg}^{2+}}$ and hence $\Delta G_{\mathrm{b}}^{0}$ are shown for the final scaling factor combination $\lambda_{\sigma,\varepsilon}^{\mathrm{RNA}}$ (Table 1, main text). R_{b} is obtained from a 100 ns straight forward simulations, the error here indicates the standard deviation of the distribution.

	$\Delta G_{\mathrm{b}}^{0}$ [k_{B}T]	$\Delta G^{\mathrm{solv}}_{\mathrm{Mg}^{2+}\rightarrow\mathrm{Ca}^{2+}}$ [k_{B}T]	$\Delta G^{\mathrm{bind}}_{\mathrm{Ca}^{2+}\rightarrow\mathrm{Mg}^{2+}}$ [k_{B}T]	R_{b} [nm]
microMg	-0.509 ±0.4	130.443 ±0.089	-126.110 ±0.088	0.2072 ±0.0043
nanoMg	-0.291 ±0.4	130.443 ±0.089	-125.892 ±0.462	0.2065 ±0.0044
exp.[45,46]	-1.036	n.a.	n.a.	0.206 - 0.208

Table S9: Binding affinity $\Delta G_{\mathrm{b}}^{0}$ obtained from backward alchemical transformation. The values given for $\Delta G^{\mathrm{solv}}_{\mathrm{Ca}^{2+}\rightarrow\mathrm{Mg}^{2+}}$ and $\Delta G^{\mathrm{bind}}_{\mathrm{Mg}^{2+}\rightarrow\mathrm{Ca}^{2+}}$ are obtained from block averaging for three blocks of 3 ns long windows each. $\Delta G^{\mathrm{bind}}_{\mathrm{Mg}^{2+}\rightarrow\mathrm{Ca}^{2+}}$ and hence $\Delta G_{\mathrm{b}}^{0}$ are shown for the final scaling factor combination $\lambda_{\sigma,\varepsilon}^{\mathrm{RNA}}$ (Table 1, main text). R_{b} is obtained from a 100 ns straight forward simulations, the error here indicates the standard deviation of the distribution.

	$\Delta G_{\mathrm{b}}^{0}$ [k_{B}T]	$\Delta G^{\mathrm{solv}}_{\mathrm{Ca}^{2+}\rightarrow\mathrm{Mg}^{2+}}$ [k_{B}T]	$\Delta G^{\mathrm{bind}}_{\mathrm{Mg}^{2+}\rightarrow\mathrm{Ca}^{2+}}$ [k_{B}T]	R_{b} [nm]
microMg	-0.469 ±0.4	-130.441 ±0.035	126.067 ±0.146	0.2072 ±0.0043
nanoMg	-0.192 ±0.4	-130.441 ±0.035	125.791 ±0.320	0.2065 ±0.0044
exp.[45,46]	-1.036	n.a.	n.a.	0.206 - 0.208

Integration of free energy profiles: In addition to the calculation of $\Delta G_{\mathrm{b}}^{0}$ via alchemical transformation, the binding affinity was obtained from integrating free energy profiles. In a first step, the binding affinity of Ca^{2+} was obtained. We started with the calculation of an one dimensional free energy profile along the Ca^{2+} - phosphate oxygen distance r_{CaOP} using Umbrella Sampling without any further restraints (Figure S5A). The window length was set to 20 ns and divided into four blocks of 5 ns each. Figure S5A shows an deep second minimum at around 0.45 nm. Moreover, the profiles show that the individual windows were not converged after 5 ns. Visual inspection confirmed the Ca^{2+} to be trapped in a direct interaction with the second phosphate oxygen of the DMP (inset in Figure S5A). Therefore, using PLUMED[29] a soft bias was

applied (eq S7) to keep the ion from interacting directly with the second oxygen (Figure S5B). Dividing the 20 ns long windows again in blocks of 5 ns each confirms that the Ca^{2+} is no longer trapped. Each of the blocks of this second free energy profile was integrated using eq S17 and subsequently averaged to calculate $\Delta G^0_{Ca^{2+}} = -4.842 \pm 0.182$ $k_B T$, similar to the value calculated for an RNA dinucleotide.[35] Similarly, the free

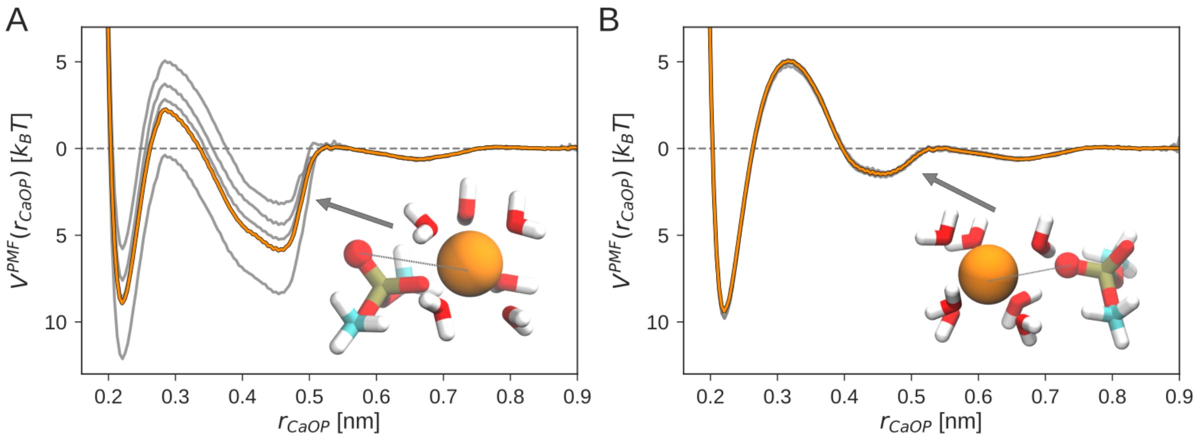

Figure S5: One dimensional potential of mean force $V^{PMF}(r_{CaOP})$ along the distance r_{CaOP} between phosphate oxygen of the DMP and Ca^{2+}. The orange curves show the profile calculated from the full window duration of 20 ns, the grey curves indicate the profiles obtained from the four blocks of 5 ns each. (A) simulated without any additional biases. (B) Additional biases are applied to keep the ion from interacting directly with the second oxygen of the phosphate groups (see section S1.5). In both cases the red sphere and dotted grey line indicate the distance to the respective phosphate oxygen at $r_{CaOP} \sim 0.45$ nm.

energy profiles for *microMg* and *nanoMg* were calculated for those scaling factors $\lambda^{RNA}_{\sigma,\varepsilon}$ obtained from the grid search and alchemical transformation calculations. The free energy profiles were obtained employing the same switching function (eq S7) to define the additional bias to keep the Mg^{2+} ion from directly interacting with the second phosphate oxygen. Integration of the resulting free energy profiles (Figure S6 and Figure 3B, main text) and block averaging over the integration of the profiles obtained from blocks of length 5 ns each results in ΔG^0_b as in Table S10.

Table S10: Binding affinity ΔG^0_b and mean distance between Mg^{2+} and the phosphate oxygen it is bound to R_b for the interaction between Mg^{2+} and the DMP. The values presented are obtained from integration of the free energy profiles shown in Figure S6.

units	ΔG^0_b [k_BT]	R_b [nm]
microMg	-0.633 ±0.6	0.2062
nanoMg	-0.375 ±0.1	0.2072
exp.[45,46]	-1.036	0.206 - 0.208

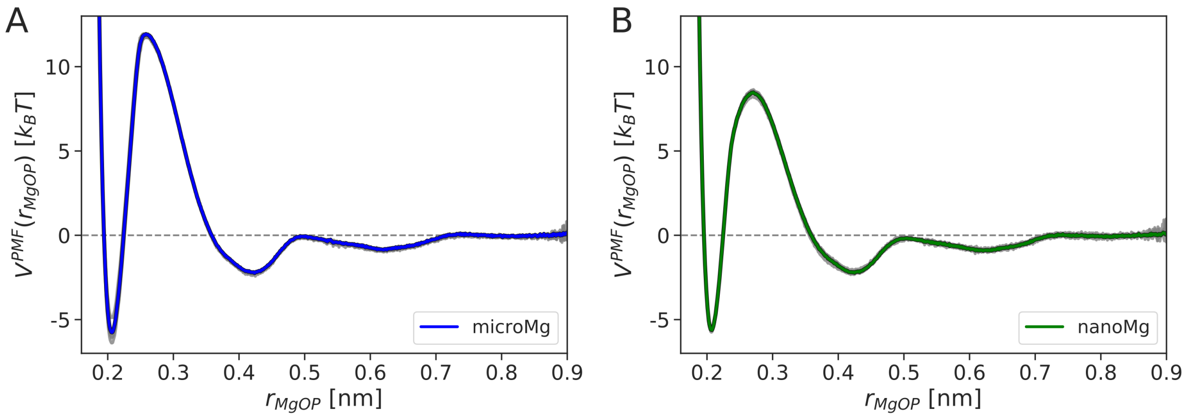

Figure S6: One dimensional potential of mean force $V^{\mathrm{PMF}}(r_{\mathrm{MgOP}})$ along the distance r_{MgOP} between phosphate oxygen of the DMP and Mg^{2+} as described by (A) *microMg* and (B) *nanoMg*. The blue and green lines show the profile calculated from the full window duration of 20 ns, the grey curves indicate the profiles obtained from the four blocks of 5 ns each. Additional biases are applied to keep the ion from interacting directly with the second oxygen of the phosphate groups (see section S1.5).

S2.5 Outer-sphere ion binding sites with *microMg* and *nanoMg*

Simulations of the *add* A-riboswitch were performed to validate the performance of both optimized force fields. The most abundant outer-sphere interaction partner for both *microMg* and *nanoMg* is the phosphate oxygen (Table S11) similar as observed with the Allnér-Villa parameter set.[36]

Table S11: Relative abundance of outer-sphere interaction partners for 100 ns long simulations of the *add* A-riboswitch. O and N specify the identity of the outer-sphere binding partners, while the indices p, b, and r indicate phosphate group, base, and ribose, respectively.

	microMg	*nanoMg*
O_p	75.5 %	76.5 %
O_b	12.9 %	11.6 %
N_b	7.0 %	7.8 %
O_r	4.6 %	4.0 %

S2.6 Identification and de novo prediction of inner-sphere binding sites with *nanoMg*

The ability of Mg^{2+} ions described by *nanoMg* to transition into inner-sphere binding on the nanosecond timescale was exploited to computationally predict Mg^{2+} binding sites. Besides the three most populated binding sites shown in the main text, additional predictions can be made (Table S12). Two more computationally predicted binding sites are shown in Figure S7A,B.

One of the five experimental binding sites has to be regarded as a crystallization artifact, as the ion is in close interaction with a crystal copy (Figure S7C). As a negative test, we can show that for this

experimental binding site as expected no overlap with any computational Mg^{2+} probability density can be observed (Figure S7C).

Table S12: Predicted binding sites for the *add* A-riboswitch. 'Populated' indicates the relative time a Mg^{2+} is bound at the specific binding site over the course of the accumulated total simulation time of 2 μs. 'Binding site' indicates the residue and which of the two phosphate oxygens the Mg^{2+} is interacting with.

Number	Populated	Binding site
(i)	67.2 %	A24:O1P
(ii)	33.2 %	A23:O2P
(iii)	31.4 %	C27:O2P
(iv)	29.2 %	A25:O2P
(v)	28.4 %	U51:O2P

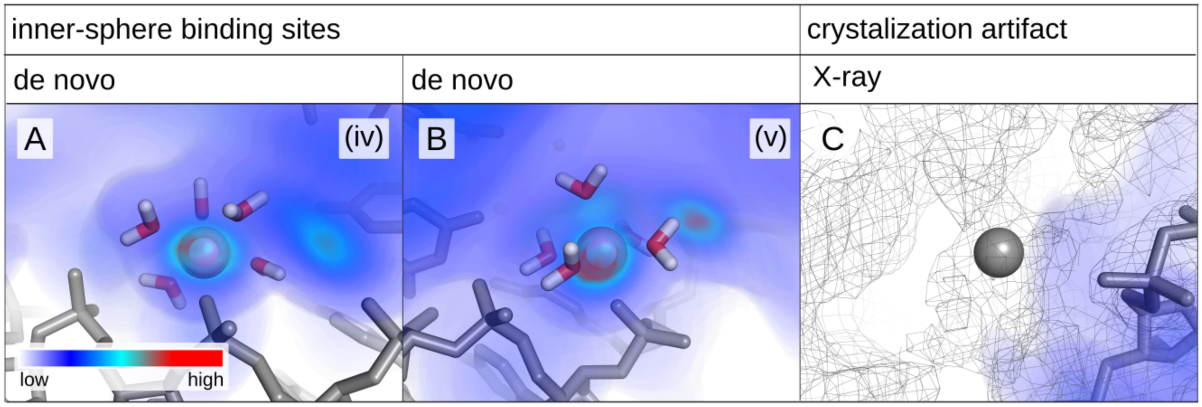

Figure S7: Representative snapshots of the *add* A-riboswitch from simulations with the *nanoMg* parameter set and three-dimensional Mg^{2+} probability density. The probability density is low in the blue regions (diffusive ions) and high in the red regions (specifically bound ions). Snapshots (iv) and (v) show the fourth and fifth most probable ion binding sites predicted from simulations with *nanoMg*. (C) The grey sphere reports the position of the Mg^{2+} that has to be regarded as a crystalization artifact. The mesh indicates the electron density obtained from X-ray crystallography.[47] The empty mesh in (C) on the left hand side of the ion corresponds to a copy of the riboswitch within the unit cell.

S3 Bibliography

(1) Berendsen, H. J.; Postma, J. P.; Van Gunsteren, W. F.; Dinola, A.; Haak, J. R. Molecular dynamics with coupling to an external bath. *J. Chem. Phys.* **1984**, *81*, 3684–3690.

(2) Hess, B. P-LINCS: A Parallel Linear Constraint Solver for Molecular Simulation. *J. Chem. Theory Comput.* **2008**, *4*, 116–122.

(3) Darden, T.; York, D.; Pedersen, L. Particle mesh Ewald: An N x log(N) method for Ewald sums in large systems. *J. Chem. Phys.* **1993**, *98*, 10089–10092.

(4) Bussi, G.; Donadio, D.; Parrinello, M. Canonical sampling through velocity rescaling. *J. Chem. Phys.* **2007**, *126*, 14101.

(5) Parrinello, M.; Rahman, A. Polymorphic transitions in single crystals: A new molecular dynamics method. *J. Appl. Phys.* **1981**, *52*, 7182–7190.

(6) Li, P.; Merz, K. M. Taking into Account the Ion-Induced Dipole Interaction in the Nonbonded Model of Ions. *J. Chem. Theory Comput.* **2014**, *10*, 289–297.

(7) Case, D. A.; Belfon, K.; Ben-Shalom, I. Y.; Brozell, S. R.; Cerutti, D. S.; Cheatham, T. E. I.; Cruzeiro, V. W. D.; Darden, T.; Duke, R. E.; Giambasu, G.; Gilson, M. K.; Gohlke, H.; Goetz, A. W.; Harris, R.; Izadi, P. A.; Izmailov, S.; Kasavajhala, K.; Kovalenko, A.; Krasny, R.; Kurtzman, T.; Lee, T. S.; LeGrand, S.; Li, P.; Lin, C.; Liu, J.; Luchko, T.; Luo, R.; Man, V.; Merz, K. M.; Miao, Y.; Mikhailovskii, O.; Monard, G.; Nguyen, H.; Onufriev, A.; Pan, F.; Pantano, S.; Qi, R.; Roe, D. R.; Roitberg, A.; Sagui, C.; Schott-Verdugo, S.; Shen, J.; Simmerling, C. L.; Skrynnikov, N. R.; Smith, J.; Swails, J.; Walker, R. C.; Wang, J.; Wilson, L.; Wolf, R. M.; Wu, X.; Xiong, Y.; Xue, Y.; York, D. M.; Kollman, P. A. Amber 2020. 2020; `https://ambermd.org/AmberTools.php`.

(8) Wang, J.; Wolf, R. M.; Caldwell, J. W.; Kollman, P. A.; Case, D. A. Development and testing of a general Amber force field. *J. Comput. Chem.* **2004**, *25*, 1157–1174.

(9) Wang, J.; Wang, W.; Kollman, P. A.; Case, D. A. Automatic atom type and bond type perception in molecular mechanical calculations. *J. Mol. Graph. and Model.* **2006**, *25*, 247–260.

(10) Vögele, M.; Köfinger, J.; Hummer, G. Molecular dynamics simulations of carbon nanotube porins in lipid bilayers. *Faraday Discuss.* **2018**, *209*, 341–358.

(11) Grotz, K.; Nueesch, M.; Holmstrom, E.; Heinz, M.; Stelzl, L.; Schuler, B.; Hummer, G. Dispersion Correction Alleviates Dye Stacking of Single-Stranded DNA and RNA in Simulations of Single-Molecule Fluorescence Experiments. *J. Phys. Chem. B* **2018**, *122*.

(12) Pérez, A.; Marchán, I.; Svozil, D.; Sponer, J.; Cheatham, T. E.; Laughton, C. A.; Orozco, M. Refinement of the AMBER force field for nucleic acids: Improving the description of α/γ conformers. *Biophys. J.* **2007**, *92*, 3817–3829.

(13) Banáš, P.; Hollas, D.; Zgarbová, M.; Jurečka, P.; Orozco, M.; Cheatham, T. E.; Šponer, J.; Otyepka, M. Performance of molecular mechanics force fields for RNA simulations: Stability of UUCG and GNRA hairpins. *J. Chem. Theory Comput.* **2010**, *6*, 3836–3849.

(14) Zgarbová, M.; Otyepka, M.; Šponer, J.; Mládek, A.; Banáš, P.; Cheatham, T. E.; Jurečka, P. Refinement of the Cornell et al. Nucleic acids force field based on reference quantum chemical calculations of glycosidic torsion profiles. *J. Chem. Theory Comput.* **2011**, *7*, 2886–2902.

(15) Bennett, C. H. Efficient estimation of free energy differences from Monte Carlo data. *J. Comput. Phys.* **1976**, *22*, 245–268.

(16) Hummer, G.; Pratt, L. R.; García, A. E.; Berne, B. J.; Rick, S. W. Electrostatic Potentials and Free Energies of Solvation of Polar and Charged Molecules. *J. Phys. Chem. B* **1997**, *101*, 3017–3020.

(17) Rami Reddy, M.; Berkowitz, M. The dielectric constant of SPC/E water. *Chem. Phys. Lett.* **1989**, *155*, 173–176.

(18) Darden, T.; Pearlman, D.; Pedersen, L. G. Ionic charging free energies: Spherical versus periodic boundary conditions. *J. Chem. Phys.* **1998**, *109*, 10921–10935.

(19) Hummer, G.; Pratt, L. R.; García, A. E. Free Energy of Ionic Hydration. *J. Phys. Chem.* **1996**, *100*, 1206–1215.

(20) Horinek, D.; Mamatkulov, S. I.; Netz, R. Rational design of ion force fields based on thermodynamic solvation properties. *J. Chem. Phys.* **2009**, *130*, 124507.

(21) Mamatkulov, S.; Schwierz, N. Force fields for monovalent and divalent metal cations in TIP3P water based on thermodynamic and kinetic properties. *J. Chem. Phys.* **2018**, *148*.

(22) Lee Warren, G.; Patel, S. Hydration free energies of monovalent ions in transferable intermolecular potential four point fluctuating charge water: An assessment of simulation methodology and force field performance and transferability. *J. Chem. Phys.* **2007**, *127*.

(23) Yeh, I.-C.; Hummer, G. System-Size Dependence of Diffusion Coefficients and Viscosities from Molecular Dynamics Simulations with Periodic Boundary Conditions. *J. Phys. Chem. B* **2004**, *108*, 15873–15879.

(24) Vega, C.; Abascal, J. L. F. Simulating water with rigid non-polarizable models: a general perspective. *Phys. Chem. Chem. Phys.* **2011**, *13*, 19663–19688.

(25) Frenkel, D.; Berend, S. *Understanding Molecular Simulation: From Algorithms to Applications*, 2nd ed.; Elsevier Science, 2001.

(26) Torrie, G. M.; Valleau, J. P. Monte Carlo free energy estimates using non-Boltzmann sampling: Application to the sub-critical Lennard-Jones fluid. *Chem. Phys. Lett.* **1974**, *28*, 578–581.

(27) Torrie, G. M.; Valleau, J. P. Nonphysical sampling distributions in Monte Carlo free-energy estimation: Umbrella sampling. *J. Comput. Phys.* **1977**, *23*, 187–199.

(28) Kumar, S.; Rosenberg, J. M.; Bouzida, D.; Swendsen, R. H.; Kollman, P. A. Multi dimensional Free -Energy Calculations Using the Weighted Histogram Analysis Method. *J. Comput. Chem.* **1994**, *16*, 1339–1.

(29) Tribello, G. A.; Bonomi, M.; Branduardi, D.; Camilloni, C.; Bussi, G. Plumed 2: New feathers for an old bird. *Comput. Phys. Commun.* **2014**, *185*, 604–613.

(30) Schwierz, N. Kinetic pathways of water exchange in the first hydration shell of magnesium. *J. Chem. Phys.* **2020**, *152*, 224106.

(31) Khavrutskii, I. V.; Dzubiella, J.; McCammon, J. A. Computing accurate potentials of mean force in electrolyte solutions with the generalized gradient-augmented harmonic Fourier beads method. *J. Chem. Phys.* **2008**, *128*.

(32) Kirkwood, J. G. The Statistical Mechanical Theory of Solutions. I. *J. Comp. Phys.* **1951**, *19*, 774.

(33) Fyta, M.; Netz, R. R. Ionic force field optimization based on single-ion and ion-pair solvation properties: Going beyond standard mixing rules. *J. Chem. Phys.* **2012**, *136*, 124103.

(34) Weerasinghe, S.; Smith, P. E. A Kirkwood-Buff derived force field for sodium chloride in water. *J. Chem. Phys.* **2003**, *119*, 11342–11349.

(35) Cruz-León, S.; Schwierz, N. Hofmeister series for metal cation-RNA interactions: The interplay of binding affinity and exchange kinetics. *Langmuir* **2020**, *36*, 5979–5989.

(36) Allnér, O.; Nilsson, L.; Villa, A. Magnesium Ion-Water Coordination and Exchange in Biomolecular Simulations. *J. Chem. Theory Comput.* **2012**, *8*, 1493–1502.

(37) Li, P.; Roberts, B. P.; Chakravorty, D. K.; Merz, K. M. Rational Design of Particle Mesh Ewald Compatible Lennard-Jones Parameters for +2 Metal Cations in Explicit Solvent. *J. Chem. Theory Comput.* **2013**, *9*, 2733–2748.

(38) Jorgensen, W. L.; Chandrasekhar, J.; Madura, J. D.; Impey, R. W.; Klein, M. L. Comparison of simple potential functions for simulating liquid water. *J. Chem. Phys.* **1983**, *79*, 926–935.

(39) Brooks, B. R.; Brooks, C. L. I.; MacKerell, A. D. J.; Nilsson, L.; Petrella, R. J.; Roux, B.; Won, Y.; Archontis, G.; Bartels, C.; Boresch, S.; Caflisch, A.; Caves, L.; Cui, Q.; Dinner, A. R.; Feig, M.; Fischer, M.; Dao, J.; Hodescek, M.; Im, W.; Kuczera, K.; Lazaridis, T.; Ma, J.; Ovchinnikov, V.; Paci, E.; Pastor, R. W.; Post, C. B.; Pu, J. Z.; Schaefer, M.; Tidor, B.; Venable, R. M.; Woodcock, H. L.; Wu, X.; Yang, W.; York, D. M.; Karplus, M. CHARMM: The Biomolecular Simulation Program. *J. Comput. Chem.* **2009**, *30*, 1545–1614.

(40) Neria, E.; Fischer, S.; Karplus, M. Simulation of activation free energies in molecular systems. *J. Chem. Phys.* **1996**, *105*, 1902–1921.

(41) Neely, J.; Connick, R. Rate of Water Exchange from Hydrated Magnesium Ion. *J. Am. Chem. Soc.* **1970**, *92*, 3476–3478.

(42) Bleuzen, A.; Pittet, P.-A.; Helm, L.; Merbach, A. E. Water exchange on magnesium(II) in aqueous solution: a variable temperature and pressure 17O NMR study. *Magn. Reson. Chem.* **1997**, *35*, 765–773.

(43) Marcus, Y. *Ion Properties*; Marcel Dekker, Inc.: New York, Basel, 1997.

(44) Tissandier, M. D.; Cowen, K. A.; Feng, W. Y.; Gundlach, E.; Cohen, M. H.; Earhart, A. D.; Coe, J. V.; Tuttle, T. R. The proton's absolute aqueous enthalpy and Gibbs free energy of solvation from cluster-ion solvation data. *J. Phys. Chem. A* **1998**, *102*, 7787–7794.

(45) Sigel, R. K.; Sigel, H. A stability concept for metal ion coordination to single-stranded nucleic acids and affinities of individual sites. *Acc. Chem. Res.* **2010**, *43*, 974–984.

(46) Leonarski, F.; D'Ascenzo, L.; Auffinger, P. Mg2+ ions: Do they bind to nucleobase nitrogens? *Nucleic Acids Res.* **2017**, *45*, 987–1004.

(47) Serganov, A.; Yuan, Y. R.; Pikovskaya, O.; Polonskaia, A.; Malinina, L.; Phan, A. T.; Hobartner, C.; Micura, R.; Breaker, R. R.; Patel, D. J. Structural basis for discriminative regulation of gene expression by adenine- and guanine-sensing mRNAs. *Chem. Biol.* **2004**, *11*, 1729–1741.
